# Enhanced infrared-to-visible upconversion imaging via metasurface–lanthanide nanoparticle hybrid screens

**DOI:** 10.1038/s41377-026-02449-5

**Published:** 2026-09-09

**Authors:** Nima Sefidmooye Azar, Matthew Parry, Xiao Qi, Changhwan Lee, Wendy S. L. Lee, Benjamin Russell, Wei Luo, Robert W. de Gille, Damian Nelson, Sivacarendran Balendhran, Jiajun Meng, Henry Tan, Gus O. Bonin, Duk-Yong Choi, P. James Schuck, Emory M. Chan, Bruce E. Cohen, Dragomir N. Neshev, Kenneth B. Crozier

**Affiliations:** 1 School of Physics, University of Melbourne, Parkville, 3010 VIC Australia; 2 ARC Centre of Excellence for Transformative Meta-Optical Systems (TMOS), University of Melbourne, Parkville, 3010 VIC Australia; 3 ARC Centre of Excellence for Transformative Meta-Optical Systems (TMOS), Research School of Physics, The Australian National University, Canberra, ACT Australia; 4 The Molecular Foundry, Lawrence Berkeley National Laboratory, Berkeley, 94720 CA USA; 5 Department of Mechanical Engineering, Columbia University, New York, 10027 NY USA; 6 Department of Materials Science and Engineering, Korea Advanced Institute of Science and Technology (KAIST), Daejeon, 34131 Republic of Korea; 7 Department of Electrical and Electronic Engineering, University of Melbourne, Parkville, 3010 VIC Australia; 8 School of Chemistry, University of Melbourne, Parkville, 3010 VIC Australia; 9 Department of Quantum Science and Technology, Research School of Physics, The Australian National University, Canberra, ACT Australia; 10 The Molecular Foundry, Division of Molecular Biophysics & Integrated Bioimaging, Lawrence Berkeley National Laboratory, Berkeley, 94720 CA USA

**Keywords:** Nanoparticles, Imaging and sensing

## Abstract

Accessing the rich information carried by infrared light typically relies on bulky, complex optoelectronic systems. Lanthanide-based upconverting nanoparticles (UCNPs) offer a compelling alternative by converting infrared light into visible photons through nonlinear anti-Stokes processes. However, achieving strong upconversion under the low excitation intensities relevant to infrared vision remains challenging, motivating strategies to enhance light–matter interaction. Here, we demonstrate enhanced infrared-to-visible upconversion imaging enabled by integrating alloyed Yb/Er UCNPs with a resonant dielectric metasurface. The metasurface supports an optical resonance aligned with the UCNP excitation band, leading to over three orders of magnitude enhancement in upconversion emission. Crucially, flat-band angular dispersion of this resonance enables uniform enhancement across incident angles relevant to imaging, thereby preserving spatial frequency content and yielding sharp, high-contrast images. In light of ongoing advances in lanthanide-based materials, this metasurface–UCNP hybrid screen provides a promising platform for compact, detector-free, and scalable infrared imaging technologies based on optical upconversion.

## Abstract (web summary)

A flat-band dielectric metasurface enhances lanthanide upconversion by over 1000×, enabling compact, high-resolution infrared-to-visible imaging.

## Graphical abstract

**Figure:**
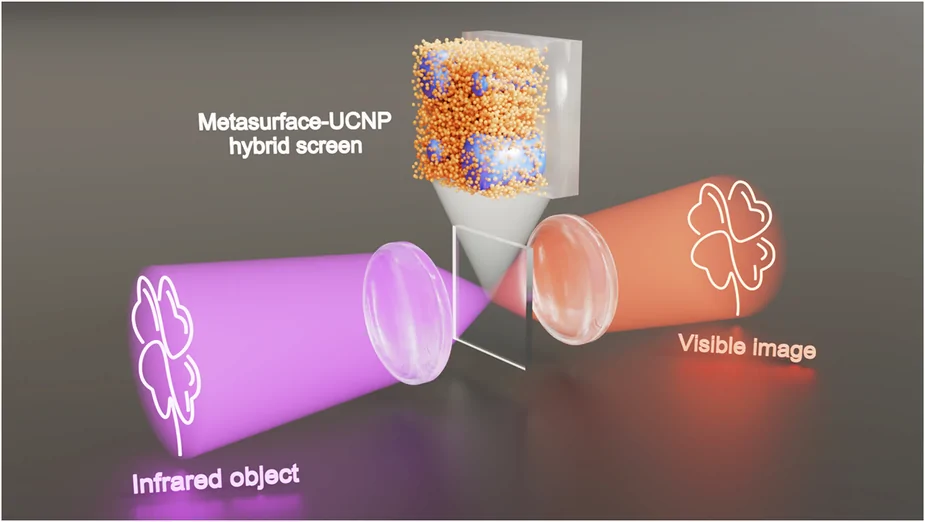

## Introduction

Infrared imaging plays a vital role in applications including environmental monitoring, industrial inspection, security, and biomedical diagnostics. However, conventional infrared imaging typically relies on narrow-bandgap optoelectronic sensors, such as InGaAs and HgCdTe, which are costly, prone to noise, and often require active cooling. These challenges have motivated growing interest in upconversion imaging, where infrared information is converted into the visible spectrum, enabling detection by the human eye and standard silicon-based detectors^1–5^.

A promising strategy for infrared-to-visible conversion is upconversion luminescence—a nonlinear process in which sequential absorption of multiple low-energy infrared photons results in the emission of a higher-energy visible photon^6^. Among various materials, lanthanide-based upconverting nanoparticles (UCNPs) have attracted significant attention due to their narrow-band emission, excellent photostability and chemical stability, and low cytotoxicity^7,8^. These unique features have enabled them to impact a broad spectrum of applications, ranging from bioimaging^9^, biosensing^10^, and therapeutic nanomedicine^11^ to super-resolution microscopy^12,13^, near-infrared^14,15^ and broadband photodetection^16,17^, photovoltaics^18,19^, and lasing^20–22^. However, the intrinsic inefficiency of multiphoton processes hinders the applicability of UCNPs under low excitation intensities^23,24^.

To address this challenge, researchers have explored integrating UCNPs with plasmonic and dielectric nanostructures that enhance light-matter interactions^25,26^. These structures improve the upconversion efficiency of UCNPs by boosting their infrared absorption^27,28^ and/or visible emission^29,30^. Localized surface plasmon resonances^31,32^, surface plasmon polaritons^33,34^, and lattice plasmons^35^ supported by metallic nanostructures can enhance excitation and emission processes in adjacent UCNPs. However, such systems often exhibit increased nonradiative losses, and in many cases the optical fields are predominantly concentrated near metal interfaces, which can limit the effective interaction volume with the UCNP layer^36,37^. Dielectric structures offer an attractive alternative, supporting low-loss resonant modes with high quality factors and more distributed field profiles. Structures such as holey photonic crystals based on silicon^38,39^ and silicon nitride^40,41^, silicon nanopillars^42^, and polymeric microresonators^20–22^ have been investigated.

Although these studies have demonstrated significant enhancements in UCNP photoluminescence, realizing high-resolution infrared-to-visible upconversion imaging remains elusive. Achieving this goal benefits from a metasurface that enhances UCNP absorption and/or emission through high-Q resonances, while simultaneously preserving the spatial frequency content of the image. High-Q resonances are often associated with nonlocal effects, whereas high-resolution imaging demands uniform absorption across spatial frequencies—a fundamentally local response. Reconciling these seemingly contradictory requirements calls for advanced dispersion engineering and novel metasurface architectures. There has been much recent interest in angle-tolerant metasurfaces for a variety of applications, ranging from terahertz devices^43^ to miniature cameras^44^ and back-reflectors^45^. To the best of our knowledge, however, their use in upconversion imaging has not been previously reported.

**Fig. 1:**
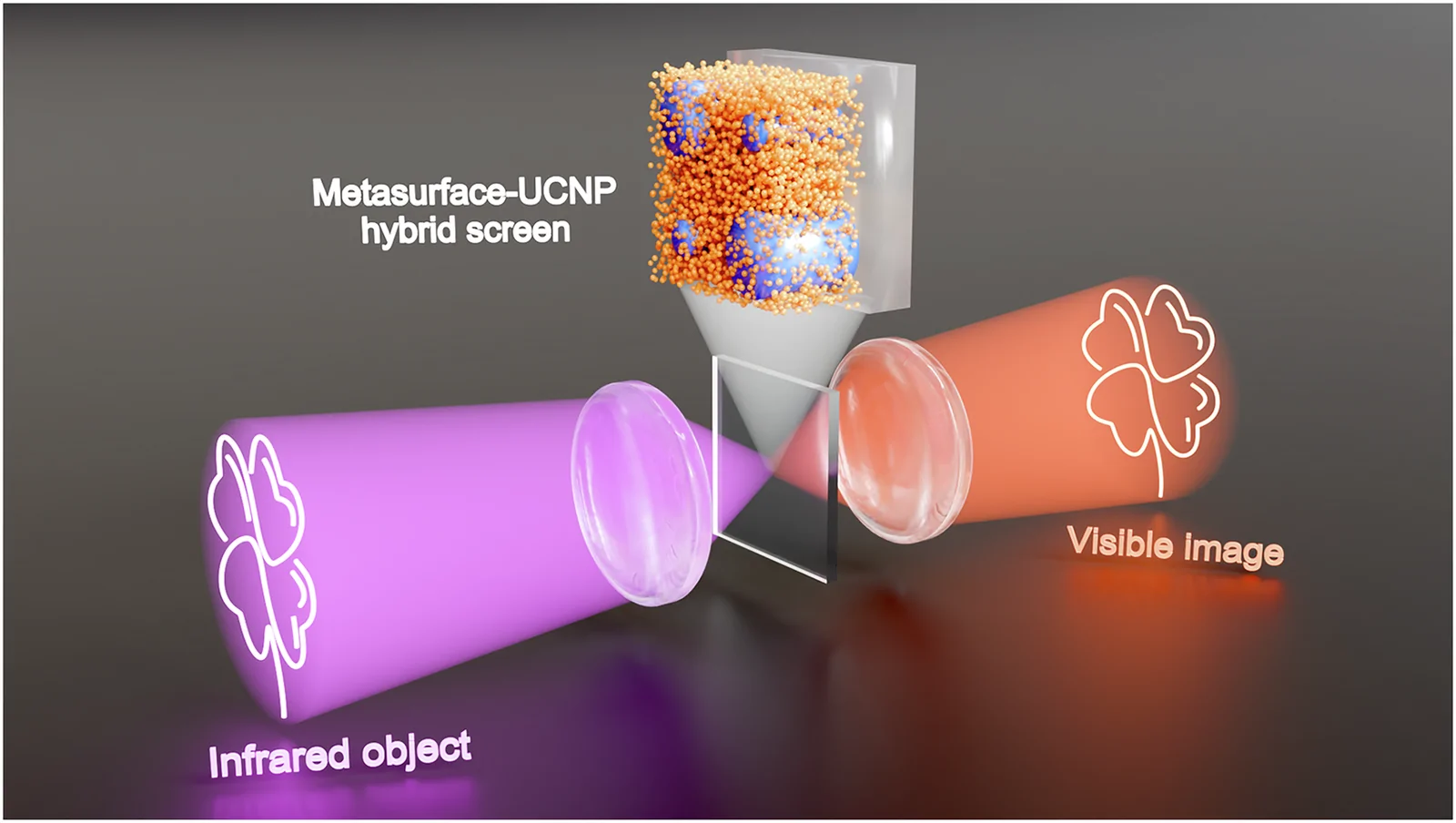
Concept of the metasurface–UCNP hybrid upconversion screen. Infrared light from an object is converted to a visible image by a UCNP layer coated on a resonant TiO₂ metasurface

In this work, we address this challenge using a symmetry-broken dielectric metasurface designed to support a flat-band, angularly robust resonance at the UCNP excitation wavelength. This resonant metasurface is integrated with a uniform UCNP coating to form a functional infrared-to-visible upconversion imaging screen, as illustrated in Fig. 1. The metasurface is optimized to enhance UCNP light absorption while maintaining minimal angular dispersion, ensuring uniform enhancement across spatial frequencies and thus preserving fine image details. Experimentally, we achieve over three orders of magnitude enhancement in upconversion emission and demonstrate high-resolution, high-contrast infrared-to-visible imaging. This platform provides a pathway toward lightweight and scalable infrared imaging systems based on optical upconversion, with potential applications ranging from infrared vision to remote sensing.

## Results

### Upconverting nanoparticles

**Fig. 2:**
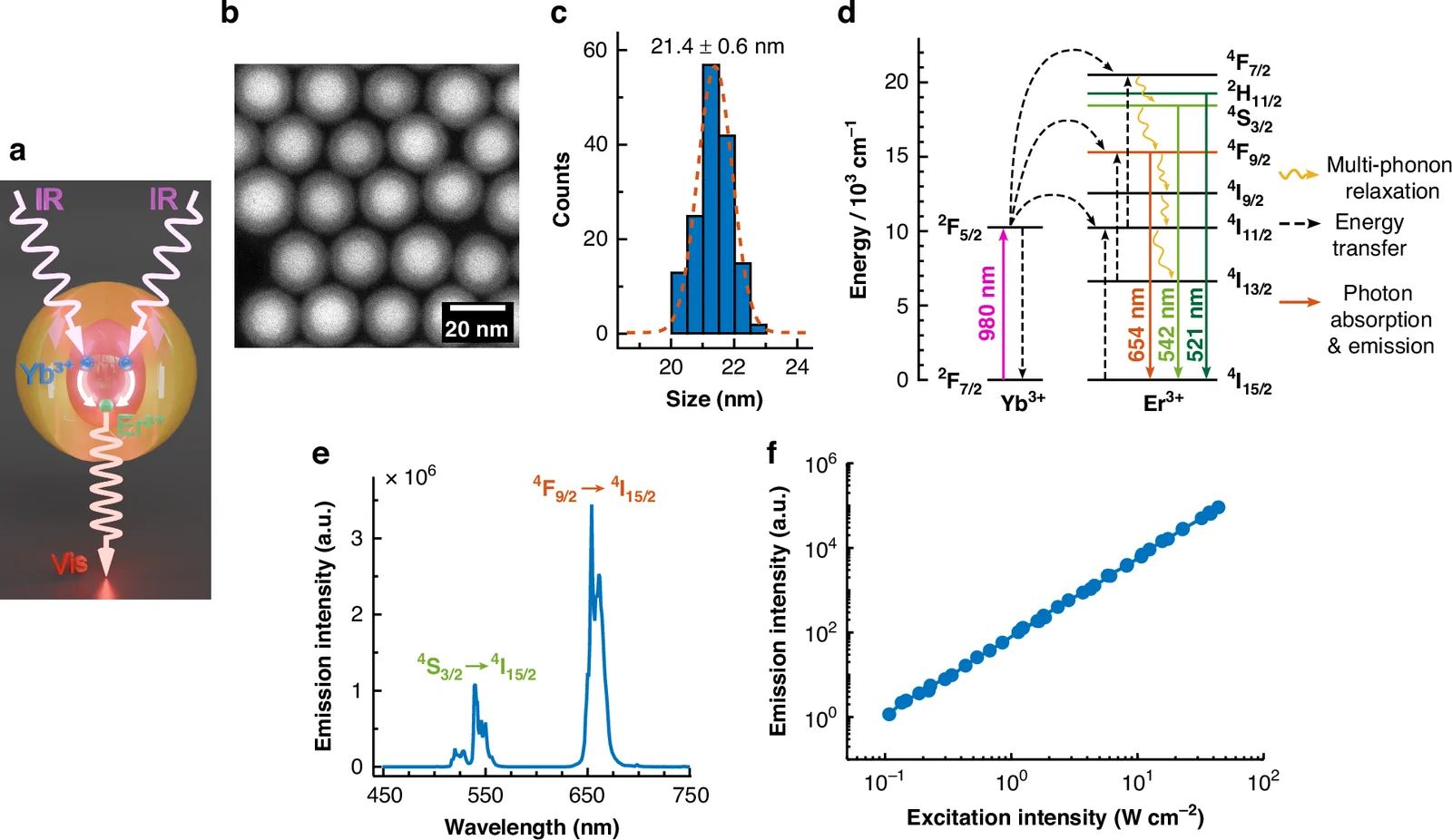
Characteristics of the lanthanide-based UCNPs. **a** Schematic illustration of a UCNP converting infrared light into visible light. **b** HAADF-STEM image of the β-phase NaYb_0.8_Er_0.2_F_4_@NaYF_4_ core-shell alloyed UCNPs. **c** Size distribution of UCNPs. **d** Energy diagram of Yb^3+^/Er^3+^ alloyed UCNPs, depicting the upconversion process. **e** Visible upconversion emission spectrum of UCNPs under 980 nm excitation at 6.6 W cm^-^², measured from a drop-cast film. **f** Log–log plot of the red emission intensity at 660 nm as a function of 980 nm excitation intensity

The key characteristics of the NaYb_0.8_Er_0.2_F_4_@NaYF_4_ alloyed UCNPs used in this study are summarized in Fig. 2. Unlike conventional UCNPs with low dopant concentrations (e.g., 20/2% Yb/Er), these alloyed UCNP cores are fully composed of Yb^3+^ and Er^3+^ ions, serving as sensitizers and emitters, respectively. The higher overall lanthanide content in alloyed UCNPs enables brighter emission, particularly under low excitation intensities^46^, which is essential for infrared-to-visible imaging. To suppress surface-related quenching, the UCNP core is encapsulated by an ~4-nm-thick undoped NaYF_4_ shell, which prevents energy migration from lanthanide ions to surface quenchers (Fig. 2a). The high-angle annular dark-field scanning transmission electron microscopy (HAADF-STEM) image (Fig. 2b) shows uniform morphology, with a mean nanoparticle size of 21.4 nm revealed by size distribution analysis (Fig. 2c). The energy-level diagram (Fig. 2d) illustrates the upconversion mechanism, where 980 nm excitation leads to red and green emission via sequential multiphoton absorption and energy transfer^47^. The corresponding emission spectrum (Fig. 2e) displays prominent peaks at 654 and 542 nm, arising from the ^4^F_9*/*2_ → ^4^I_15*/*2_ and ^4^S_3*/*2_ → ^4^I_15*/*2_ transitions of Er^3+^, respectively. The nonlinear excitation dependence of the red emission is shown in Fig. 2f as a log–log plot of the 660 nm intensity versus 980 nm excitation intensity. Linear fitting yields a power-law dependence *I* ∝ *P*^*n*^ with a slope of *n* ≈ 1.9, indicating a predominantly two-photon upconversion process for the red emission under the investigated excitation regime.

### Fabrication of the metasurface-UCNP hybrid screen

**Fig. 3:**
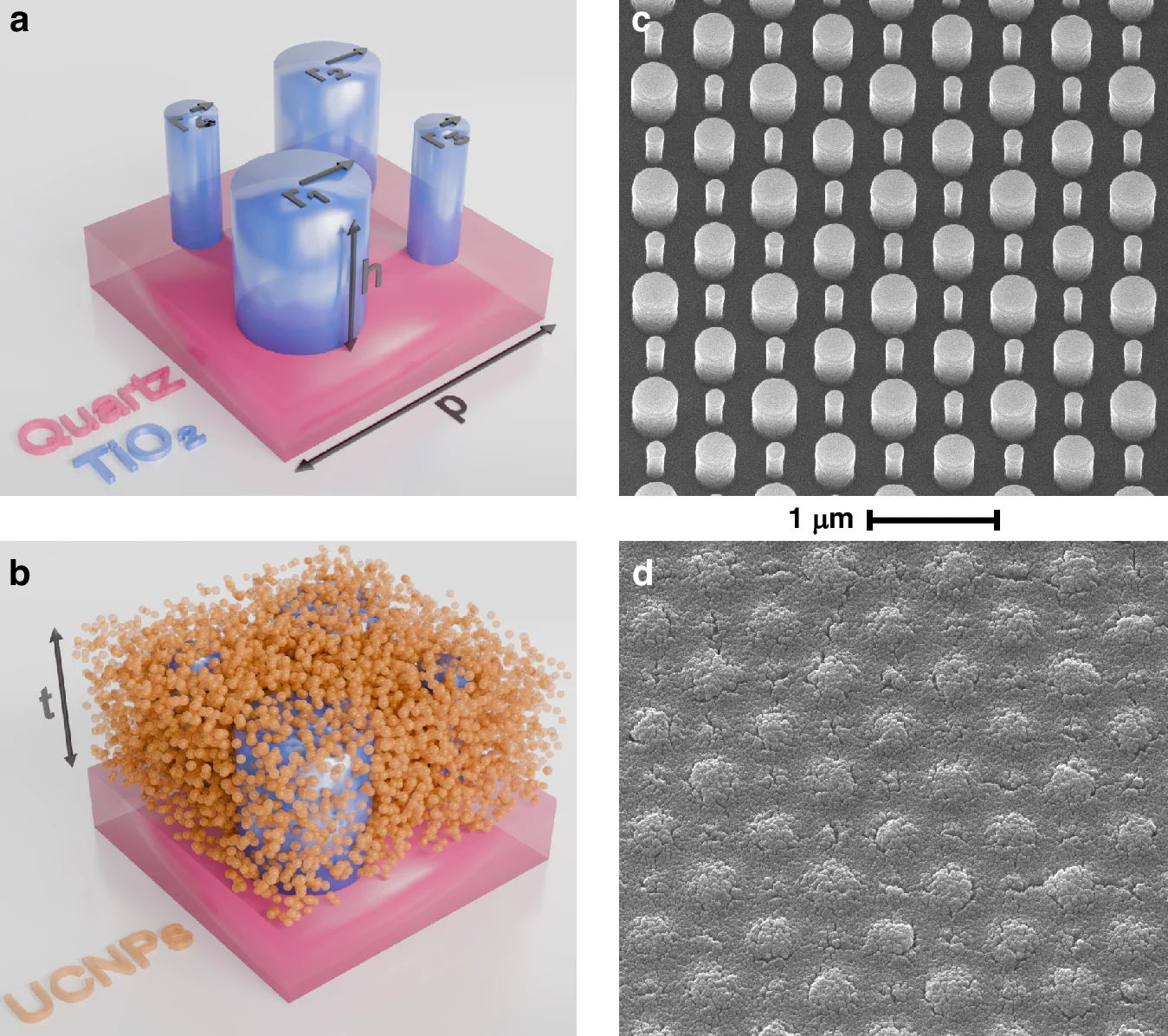
The metasurface-UCNP screen. **a**, **b** Schematic representation of the metasurface unit cell before and after spin-coating with UCNPs, respectively. **c**, **d** Tilted (30˚) SEM images of the metasurface before and after UCNP deposition, respectively

To enhance light-matter interaction and thereby improve upconversion efficiency, UCNPs were integrated with a titanium dioxide (TiO_2_) metasurface designed to support optical resonances at their excitation and emission bands. Figure 3 presents schematic illustrations and scanning electron microscope (SEM) images of the metasurface before and after UCNP deposition. The metasurface unit cell comprises four TiO_2_ nanopillars on a quartz substrate (Fig. 3a, c). The TiO_2_ metasurface was fabricated following the procedure described in ref. ^48^ and illustrated in Fig. S1. The geometrical parameters were designed as follows: period, *p* = 900 nm; pillar radii, *r*_1_ = 163 nm, *r*_2_ = 147 nm, *r*_3_ = *r*_4_ = 70 nm; and pillar height, *h* = 375 nm. A uniform UCNP layer, with thickness *t* = 425 nm, was deposited by spin-coating the nanoparticle solution onto the metasurface (Fig. 3b, d).

### Design of the metasurface-UCNP screen

The metasurface employs an asymmetric architecture based on a band-folding strategy^49,50^. The symmetry of the square lattice of nanopillars is broken by assigning them unequal radii within the unit cell, which doubles the lattice period and folds modes from the M-point of the original Brillouin zone to the Γ-point of the symmetry-broken lattice. These folded modes correspond to quasi-bound states in the continuum (quasi-BIC), which arise from symmetry-protected modes that become weakly radiative upon symmetry breaking. Unlike conventional Mie resonances that are primarily confined within high- index resonators, these quasi-BIC modes exhibit strong field localization in the surrounding medium, enabling enhanced interaction with the UCNP layer. Furthermore, symmetry breaking induces mode interactions that lead to flat-band dispersion near the Γ-point. Following these principles, we optimized the metasurface to support resonances aligned with the excitation and emission bands of UCNPs. Specifically, we maximize the figure of merit, FOM = (*abs*_975_ × *abs*_660_)/*t*^2^. Here, *abs*_975_ and *abs*_660_ represent the simulated light absorption in the UCNP layer, in the presence of the metasurface, at *λ* = 975 nm (corresponding to the excitation laser and the UCNP excitation band) and *λ* = 660 nm (corresponding to the primary UCNP emission band), respectively. *t* denotes the thickness of the UCNP film. This FOM, intended to capture the upconversion enhancement provided by the metasurface, places equal emphasis on excitation and emission processes and intentionally disregards the nonlinear, multiphoton nature of excitation, thereby promoting multi-resonant structures. Light absorption serves as a proxy for light-matter interaction, and by the reciprocity principle, *abs*_660_ is proportional to the emission intensity at *λ* = 660 nm. Normalization by *t*^2^ accounts for the approximate linear scaling of *abs*_975_ and *abs*_660_ with UCNP film thickness in the absence of the metasurface. The nanopillar radii *r*_3_ and *r*_4_ were set equal to ensure polarization independence under normal incidence. Other optimization constraints and parameter ranges are listed in Table S1. Multiple optimization runs were conducted with randomly initialized parameters. The designs with the best calculated performance were fabricated, and the sample demonstrating the best measured performance is reported in this article.

**Fig. 4:**
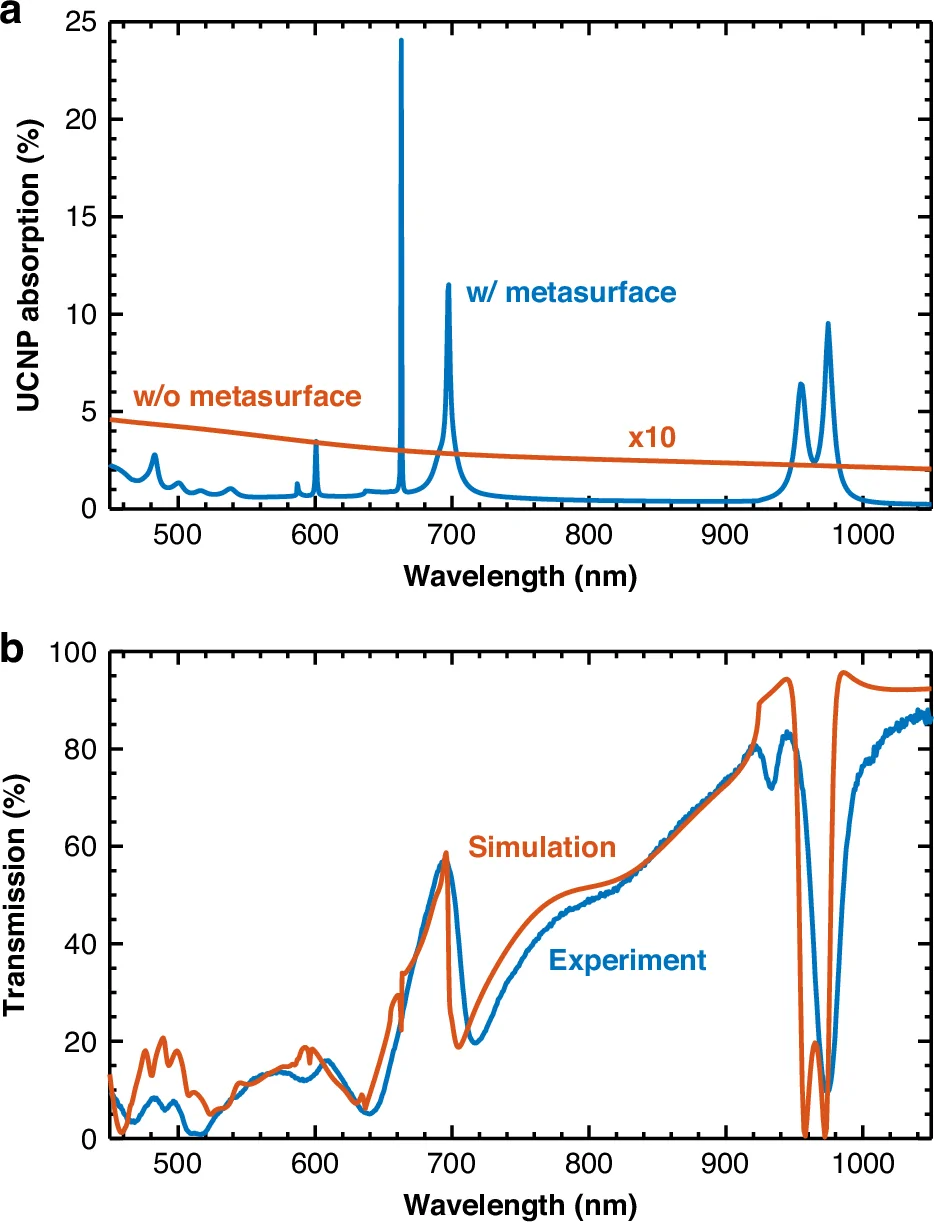
Design of the dielectric metasurface. **a** Simulated UCNP light absorption spectra with and without the metasurface. **b** Simulated zeroth-order transmission spectrum of metasurface–UCNP screen, compared with experimental measurement

The simulated light absorption in the UCNP film and the transmission spectra of the upconversion screen are presented in Fig. 4. This design features a relatively broad resonance at the UCNP excitation band and a sharper resonance at the emission band, enabling enhanced light–matter interaction at both excitation and emission wavelengths (Fig. 4a). Using the experimentally measured nonlinear power-law dependence *I* ∝ *P*^*n*^ with *n* ≈ 1.9, the expected upconversion enhancement (Enh.) can be estimated as (*abs*_975_ Enh.)^1.9^ x (*abs*_660_ Enh.). From the simulated absorption enhancement factors, the excitation contribution is 43.1^1.9^ ≈ 1,275, while the emission contribution is approximately 80. The resulting total enhancement is therefore estimated to be 1.02 × 10^5^. This value represents an upper bound as it assumes perfect spatial overlap between the excitation and emission resonant modes. It should also be noted that the absorption and enhancement values depend on the extinction coefficient of the UCNP film, which is assumed to be *k* = 0.0005 in this study. Comparison of calculated and measured transmission spectra (Fig. 4b) reveals that the simulation accurately predicts the resonance at *λ* = 975 nm and shows good qualitative agreement with the experimental data. However, the fabricated device lacks the expected resonance at *λ* = 660 nm. Further analysis indicates that this resonance is sensitive to the refractive index of the UCNP film (Fig. S2). Therefore, this discrepancy is likely due to inaccuracies in the optical constants used in the simulation.

**Fig. 5:**
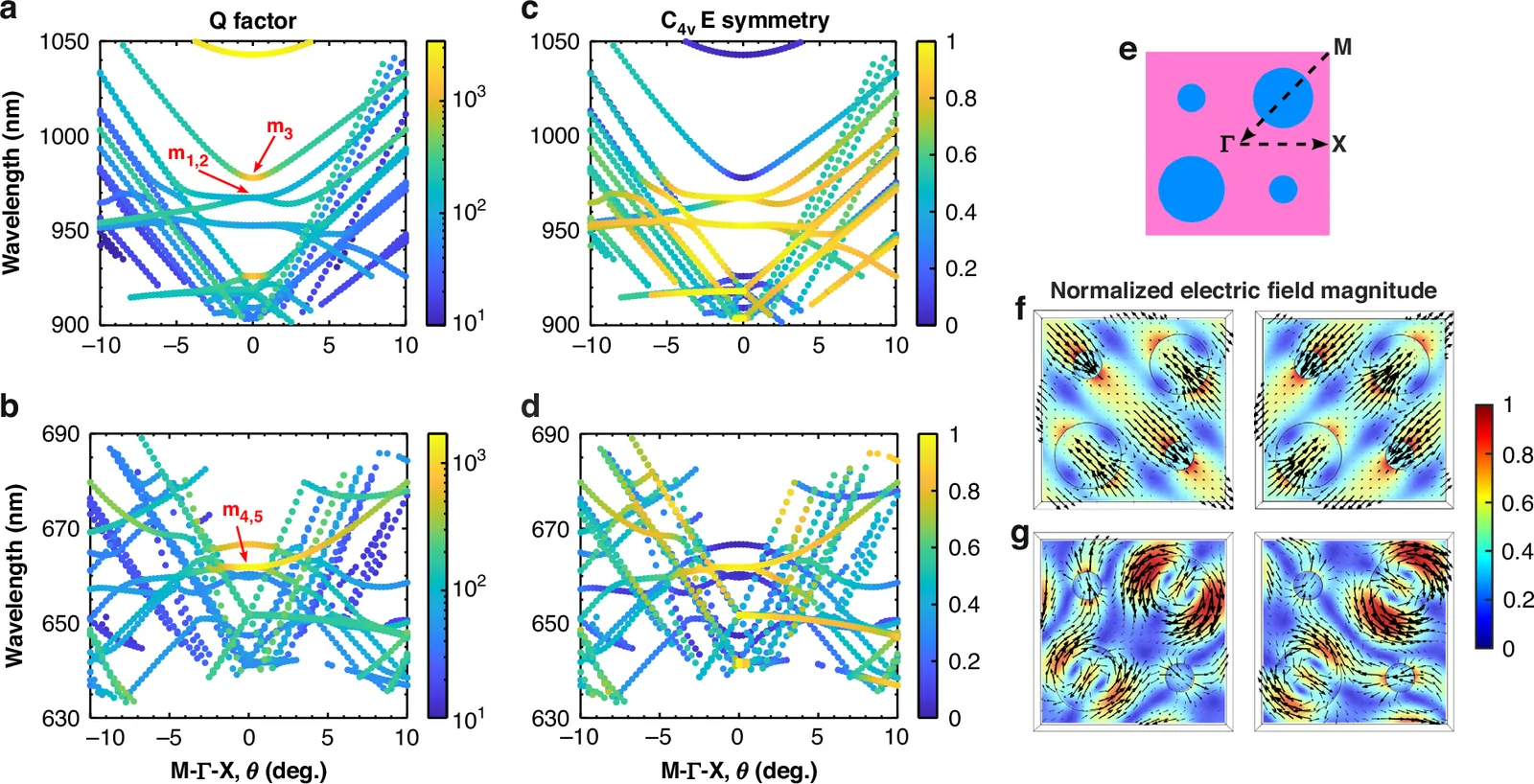
Optical mode analysis of the UCNP-coated dielectric metasurface. **a**, **b** Simulated Q factor of the metasurface modes around the UCNP excitation and emission bands, respectively. **c**, **d** Symmetry analysis of these modes with respect to the C_4v_ E irreducible representation, highlighting bright modes that can be excited by plane wave illumination. **e** Schematic showing the M-Γ-X direction corresponding to (**a**–**d**). **f** Electric field distributions of the two degenerate bright modes at the excitation band, labeled m_1,2_ in (**a**). **g** Electric field distributions of the two degenerate bright modes at the emission band, labeled m_4,5_ in panel b. The fields are shown on a horizontal plane intersecting the mid-height of the nanopillars

To gain deeper insight into the photonic modes supported by the metasurface and their potential role in the upconversion enhancement, eigenmode and symmetry analysis were performed. Figure 5 presents the simulated Q factors, symmetry characteristics, and electric field distributions of the modes supported by the UCNP-coated dielectric metasurface. The structure supports multiple high-Q resonances in the vicinity of both the UCNP excitation (Fig. 5a) and emission (Fig. 5b) bands, which are of interest for enhancing light–matter interactions. Symmetry analysis, carried out using projection operators onto the high-symmetry modes of the metasurface^50^, further elucidates which modes are relevant for experimental excitation and emission. The metasurface exhibits C_4v_ symmetry, characterized by four mirror planes—horizontal, vertical, and two diagonals—intersecting at the center of each resonator. Eigenmodes are decomposed with respect to the C_4v_ E irreducible representation (Fig. 5c, d) to identify bright modes capable of coupling to linearly polarized free-space light—an essential requirement for efficient experimental excitation and collection, and thus a key design criterion for upconversion enhancement. Two pairs of degenerate modes, labeled m_1,2_ at the excitation band and m_4,5_ at the emission band, exhibit strong C_4v_ E symmetry and high Q factors. Their corresponding electric field distributions (Fig. 5f, g) show pronounced field confinement within the UCNP layer, particularly at the interface with the TiO_2_ nanopillars. This field distribution is a key distinction from both conventional dielectric Mie resonances and plasmonic resonances. In Mie-type dielectric resonators, the field is primarily confined within the high-index material, whereas in plasmonic systems the field is strongly localized near metal interfaces and may be accompanied by increased nonradiative losses. In contrast, the quasi-BIC modes employed here provide strong field enhancement within the UCNP layer while maintaining low optical loss, thereby enabling efficient upconversion enhancement. These bright, spatially overlapping resonances are well-suited to simultaneously enhance excitation and emission processes in UCNPs. However, not all high-Q modes contribute effectively. The mode m_3_, for example, shows a weak projection onto the C_4v_ E symmetry, indicating that it cannot be excited by linearly polarized plane waves. Further analysis attributes this mode to the C_4v_ A_2_ irreducible representation (Fig. S3a), i.e., a dark mode, and the associated electric field distribution (Fig. S3b) exhibits a vortex-like transverse pattern.

The flat dispersion of the modes m_1,2_ near the Γ-point, arising from band interactions, is also noteworthy, as it ensures angle-insensitive resonance behavior—highly beneficial for imaging applications. The Brillouin zone folding (BZF) mechanism that produces flat bands can be understood in physically intuitive terms^43^. In BZF, a photonic band originally located at the edge of the Brillouin zone is translated to the Γ-point by enlarging the unit cell. At the Brillouin zone boundary, the group velocity ${v}_{g}=\partial \omega /\partial {k}$ vanishes due to symmetry, meaning the dispersion slope is zero. When this band is folded to Γ, the zero first-order derivative is preserved, resulting in a locally flat dispersion near normal incidence. Although the flat region is limited in k-space extent, this reduced angular dispersion is sufficient to enhance angular robustness around the Γ-point. In summary, the presence of high-Q resonances at the excitation and emission bands, along with their favorable symmetry, field confinement, and dispersion characteristics, underpins the metasurface’s capacity to enhance upconversion efficiency.

### Optical characterization of the metasurface-UCNP screen

**Fig. 6:**
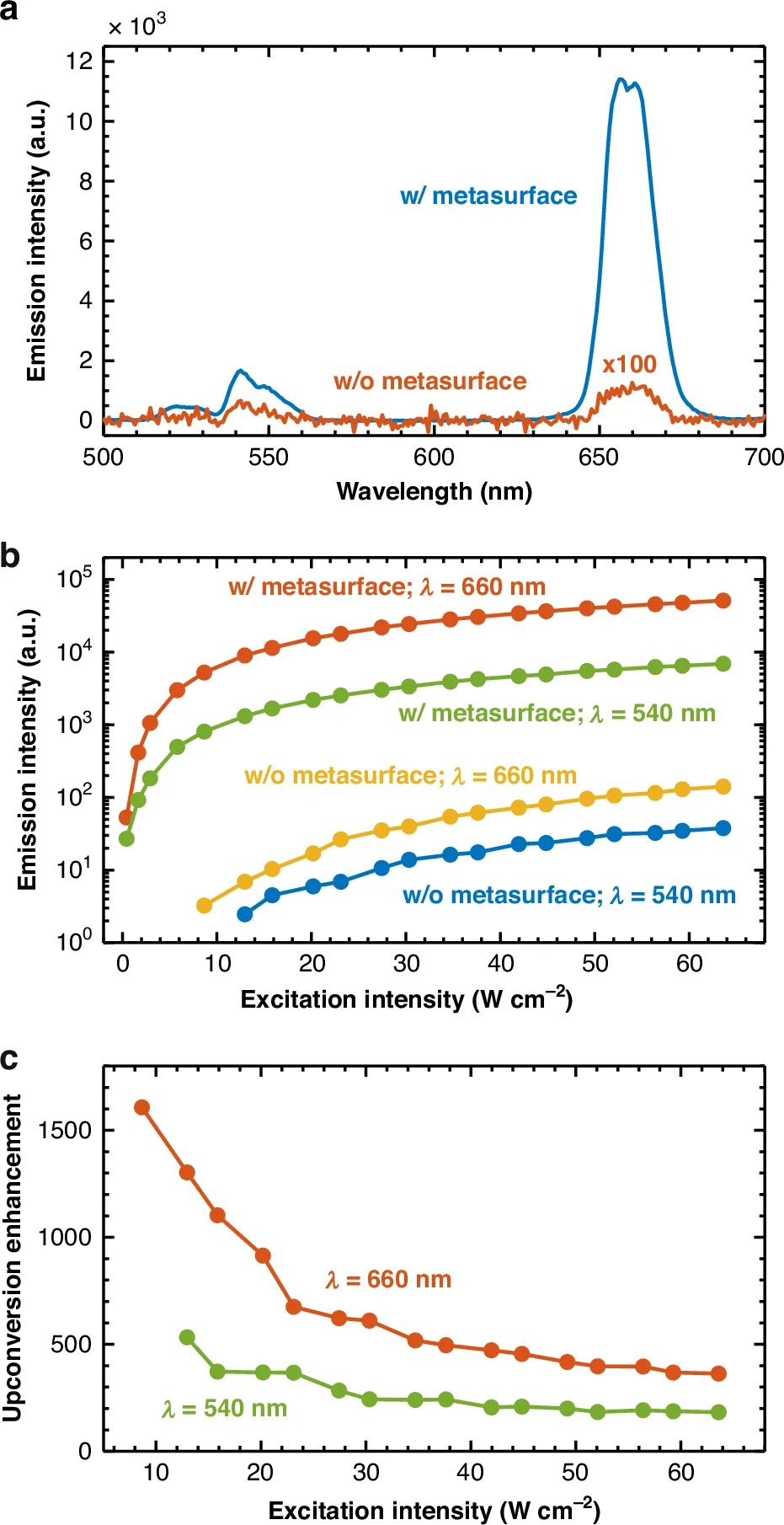
Upconversion characteristics of the metasurface–UCNP screen. **a** Measured emission spectra with and without the metasurface (the latter scaled by a factor of 100 to facilitate comparison) at an excitation intensity of 15.9 W cm^-2^. **b** Emission intensity at *λ* = 660 nm and 540 nm as a function of excitation intensity with and without the metasurface. **c** Upconversion enhancement provided by the metasurface as a function of excitation intensity at *λ* = 660 and 540 nm. Excitation wavelength is 976 nm

Figure 6 shows the experimental characterization of the metasurface–UCNP system, comparing the upconversion emission with and without the metasurface. The emission spectrum (Fig. 6a) reveals a significant enhancement of both red and green emission bands. The dependence of the upconversion emission intensity on excitation power at *λ* = 660 and 540 nm, and the corresponding upconversion enhancement factor—defined as the ratio of emission with and without the metasurface— were further studied (Fig. 6b, c). The upconversion enhancement is especially pronounced at low excitation intensities and saturates at higher excitation powers, underscoring the metasurface’s effectiveness in boosting upconversion efficiency under weak pumping conditions. The metasurface provides an upconversion enhancement of 1100-fold at λ = 660 nm under an excitation intensity of 15.9 W cm^−2^. This performance is comparable to the highest enhancement factors reported for metasurface–UCNP and plasmonic–UCNP hybrid structures (Table S2).

As discussed earlier, the fabricated screen appears to lack the intended resonance at the emission band, potentially due to inaccuracies in the refractive index values used in the design process. To further explore this discrepancy, the angular and temporal emission characteristics of the screen were investigated. Figure S4 illustrates how the metasurface influences the directionality of upconverted emission in theory and practice. Simulation of angle-resolved absorption (Fig. S4a) on the emission band resonance reveals strong directional behavior, which becomes less directional and more spatially distributed under off-resonance conditions (i.e., a few nanometers detuned from resonance). In contrast, experimental back focal plane images of the red emission at various excitation intensities (Fig. S4b) do not exhibit directional emission. Furthermore, time-resolved measurements, shown in Fig. S5, demonstrate that the presence of the metasurface does not alter the emission decay kinetics of the UCNPs under pulsed excitation. Both the metasurface and reference samples exhibit an identical lifetime of 284 µs. These findings suggest that the observed enhancement in upconversion is primarily due to the excitation band resonance. To achieve simultaneous enhancement at both the excitation and emission bands—as predicted by simulations—a more accurate characterization of the materials’ optical constants is necessary. This could enable significantly greater upconversion enhancement, directional emission, and potentially lasing.

### Infrared-to-visible upconversion imaging

**Fig. 7:**
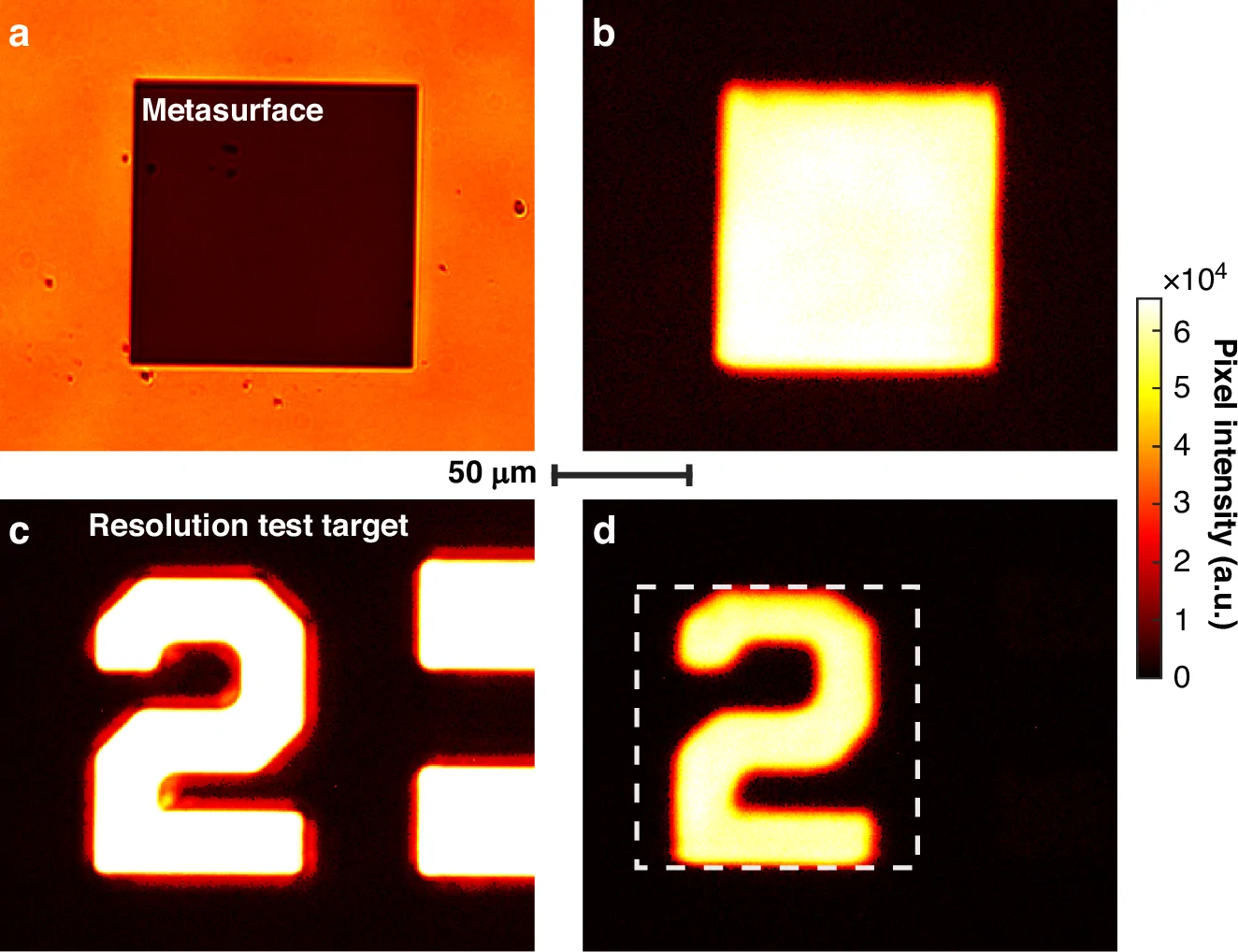
Upconversion imaging using the metasurface–UCNP screen. **a** Image of the screen under broadband illumination. **b** Upconverted image of the screen under infrared excitation. **c** Image of the object under broadband illumination. **d** Upconverted image of the object under infrared excitation, with the patterned metasurface area indicated by the white dashed square. The infrared light source has a wavelength of *λ* = 976 nm and an intensity of 7.3 W cm^−2^. Upconverted images are captured after a 750-nm short-pass filter

Finally, infrared-to-visible upconversion imaging is demonstrated using the metasurface-enhanced UCNP screen, as shown in Fig. 7. The patterned metasurface region is visible under broadband illumination (Fig. 7a) and emits bright upconverted light under infrared excitation (Fig. 7b). To evaluate imaging performance, a resolution target was placed in the optical path and imaged onto the upconversion screen. The object is shown under broadband illumination without the upconversion screen (Fig. 7c) and under infrared excitation with the screen in place (Fig. 7d). The white dashed square marks the metasurface-patterned area, which exhibits substantially stronger upconversion emission than the surrounding unpatterned regions, confirming the metasurface’s enhancement effect. Importantly, the upconverted image preserves fine spatial details, indicating high-resolution infrared-to-visible imaging.

**Fig. 8:**
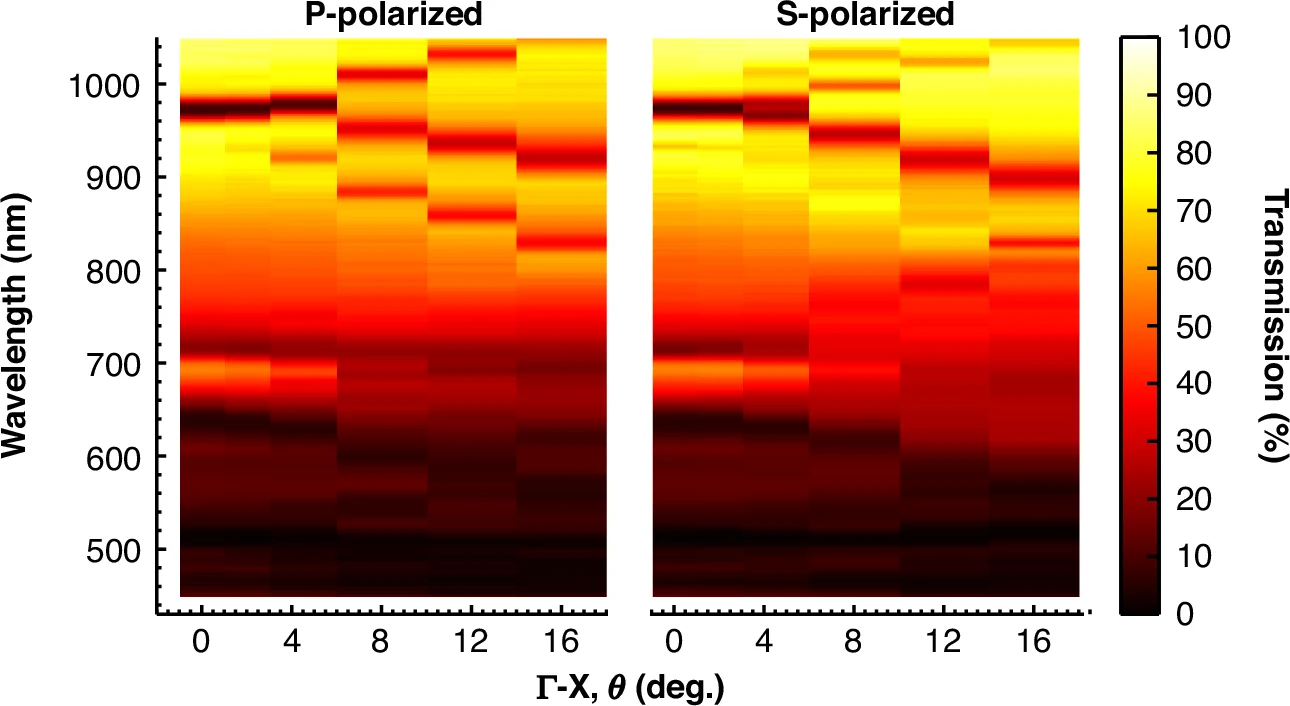
Angle-resolved transmission of the metasurface–UCNP screen. Transmission spectra as a function of incident angle under p-polarized and s-polarized illumination

This capability arises from the flat-band angular dispersion of the metasurface resonance, which ensures uniform upconversion enhancement across incident angles and therefore across spatial frequencies. Angle-resolved transmission measurements (Fig. 8) confirm that the resonance at the UCNP excitation band remains invariant with respect to both polarization and incident angle up to ~6°. The support of polarization-independent and angularly robust modes is crucial for maintaining image fidelity and is particularly advantageous for real-world applications involving unpolarized, diffuse illumination.

To quantitatively evaluate spatial resolution preservation, an edge-based modulation transfer function (MTF) analysis was performed on both the visible reference image and the upconverted image recorded at the camera plane (see Supplementary Information and Fig. S6). After accounting for the 20× magnification of the 0.4 NA objective, the extracted MTF50 values correspond to 0.20 cycles µm^−1^ for the visible image and 0.10 cycles µm^−1^ for the upconverted image at the metasurface plane. This indicates a reduction in high-spatial-frequency contrast by approximately a factor of two following upconversion. The metasurface angular bandwidth (±6°) theoretically supports spatial frequencies up to ~0.16 cycles µm^−1^ at the emission wavelength, which is comparable to the measured upconverted bandwidth. In contrast, the diffraction limit of the objective (0.4 NA) and the detector sampling limit are substantially larger and therefore do not constrain the observed resolution. The measured reduction in MTF50 likely arises from a combination of relay optics degradation and the finite angular bandwidth of the metasurface. Importantly, spatial frequencies associated with ~20 µm features of the object (0.05 cycles µm^−1^) lie below the measured upconverted bandwidth (~0.10 cycles µm^−1^), explaining the preservation of these features in the infrared-to-visible images.

## Discussion

We have presented a metasurface-based strategy for substantially enhancing the upconversion efficiency of lanthanide-based nanoparticles while enabling high-resolution infrared-to-visible imaging. The TiO_2_ metasurface, designed to support multiple optical resonances aligned with the excitation and emission bands of UCNPs, achieves a significant enhancement of light–matter interaction. Mode analysis revealed the presence of bright, high-Q, and spatially overlapping resonances at these bands. Although the fabricated structure did not reproduce the designed resonance at the emission band—likely due to inaccuracies in the refractive index data—it still achieved over three orders of magnitude enhancement in red upconversion emission under low-intensity infrared excitation, attributed to the excitation band resonance. Notably, the metasurface supports a flat-band angular dispersion at the excitation wavelength, ensuring uniform enhancement across spatial frequencies and preserving fine image details. This capability was experimentally validated through high-resolution infrared-to-visible imaging, where sharp, high-contrast images were obtained.

The imaging demonstration was performed at an excitation intensity of 7.3 W cm^−2^ to ensure sufficient signal-to-noise ratio for quantitative spatial resolution analysis. This excitation level is substantially higher than typical ambient illumination (e.g., ~0.1 W cm^−2^ for sunlight and significantly lower for indoor lighting), and therefore the present implementation represents actively excited upconversion imaging rather than ambient-light operation. Importantly, as shown in Fig. 6b, the metasurface-integrated UCNP layer produces clearly distinguishable emission at excitation intensities as low as 0.4 W cm^−2^, whereas the bare UCNP film requires approximately 9 W cm^−2^ to generate distinguishable visible emission. This reduction in required excitation intensity highlights the enhancement capability of the metasurface. Continued improvements in UCNP quantum efficiency and metasurface engineering may enable operation at substantially lower excitation levels in future implementations.

While the devices in this study were fabricated using electron-beam lithography to ensure precise control of the metasurface geometry, the planar dielectric design is fully compatible with scalable, large-area fabrication approaches such as nanoimprint lithography, which are widely used for wafer-scale metasurface production^51^.

By combining strong upconversion enhancement, angular robustness, and polarization independence, this metasurface–UCNP platform provides a promising foundation for next-generation compact and scalable upconversion-based infrared imaging technologies compatible with standard silicon-based visible cameras.

## Materials and methods

### Simulations

The rigorous coupled-wave analysis (RCWA) method and the particle swarm optimization algorithm, implemented in the Ansys Lumerical software, were employed to compute the figure of merit and to design the metasurface. The light absorption and transmission spectra were also calculated using RCWA. Eigenmode analysis results, as well as the angular distribution of UCNP absorption, were obtained using COMSOL Multiphysics. The wavelength-dependent complex refractive index values used in the optical simulations are shown in Fig. S7. For TiO_2_, *n*(*λ*) and *k*(*λ*) were measured via spectroscopic ellipsometry of a 270-nm-thick film deposited on a Si substrate. For the UCNP film, *n*(*λ*) was measured via ellipsometry of a 425-nm-thick film spin-coated on a quartz substrate. The extinction coefficient *k* was below the measurement sensitivity and was therefore approximated as a small constant value (*k* = 0.0005) in the simulations to evaluate the absorption-based figure of merit.

### Synthesis of UCNPs

β-phase alloyed NaYb_0.8_Er_0.2_F_4_ nanocrystals, 14 nm in diameter, were synthesized using previously reported procedures with minor modifications^52^. Subsequently, 4-nm-thick epitaxial NaYF_4_ inert shells were grown on the nanocrystals by optimizing a successive ion layer adsorption and reaction (SILAR) approach in a laboratory automation robot, the Workstation for Automated Nanocrystal Discovery and Analysis (WANDA)^53^. Detailed procedures are provided in the Supplementary Information.

### Fabrication of the metasurface-UCNP screen

The UCNP thin film was integrated with the TiO_2_ metasurface via spin coating to create a uniform upconversion screen. Details of spin-coating are provided in the Supplementary Information.

### Characterization of the metasurface-UCNP screen

Upconversion and transmission measurements were performed using the Köhler illumination setup illustrated in Fig. S8. An infrared laser (*λ* = 976 nm) and a broadband lamp served as the light sources for the upconversion and transmission measurements, respectively. Objective lenses OL_1_ and OL_2_ were used to collimate the beams. Mirror M_1_ directed the selected source beam through aperture A_1_, which defined the illumination area. Aperture A_2_ was used to control the angular spread of the incident beam. The objective lens OL_3_ focused the light onto the upconversion (UC) screen, and OL_4_ collected the emitted or transmitted light. The beam was then passed through lens L_1_, which focused it onto aperture A_3_ to define the observation area. Lens L_2_ collected the spatially filtered light, and a 750-nm short-pass filter (SPF) was used to block the excitation infrared light during upconversion measurements. Mirror M_2_ directed the beam toward a Si camera for visualizing the measurement area, with lens L_3_ projecting the image onto the camera sensor. Alternatively, the objective lens OL_5_ focused the light into an optical fiber connected to a spectrometer for spectral analysis. The object was added to the setup, along with several minor modifications, as shown in Fig. S9, to perform upconversion imaging. The same setup was further adapted, as illustrated in Fig. S10, to capture back focal plane images. A 650/40-nm band-pass filter (BPF) was introduced to isolate the red emission.

## Supplementary information

Supplementary Information

## Data Availability

The data that support the findings of this study are available from the corresponding author upon reasonable request.
