# Supplementary material for: Enhanced infrared-to-visible upconversion imaging via metasurface–lanthanide nanoparticle hybrid screens
Source: Light Sci Appl. 2026 Sep 9;15:377. doi: 10.1038/s41377-026-02449-5 (PMC13558671; doi:10.1038/s41377-026-02449-5)
Supplement: Supplementary file 1 [file 41377_2026_2449_MOESM1_ESM.pdf]

Supplementary Information for

Enhanced infrared-to-visible upconversion imaging via metasurface–lanthanide nanoparticle hybrid screens

Nima Sefidmooye Azar[1,2], Matthew Parry[3], Xiao Qi[4], Changhwan Lee[5,6], Wendy S. L. Lee[2,7], Benjamin Russell[1,2], Wei Luo[8], Robert W. de Gille[1], Damian Nelson[1], Sivacarendran Balendhran[1], Jiajun Meng[1,2], Henry Tan[1,2], Gus O. Bonin[1], Duk-Yong Choi[9], P. James Schuck[5], Emory M. Chan[4], Bruce E. Cohen[4,10], Dragomir N. Neshev[3], and Kenneth B. Crozier[1,2,7]*

[1]School of Physics, University of Melbourne, Parkville, Victoria 3010, Australia

[2]ARC Centre of Excellence for Transformative Meta-Optical Systems (TMOS), University of Melbourne, Parkville, Victoria 3010, Australia

[3]ARC Centre of Excellence for Transformative Meta-Optical Systems (TMOS), Research School of Physics, The Australian National University, Canberra, Australia

[4]The Molecular Foundry, Lawrence Berkeley National Laboratory, Berkeley, California 94720, United States

[5]Department of Mechanical Engineering, Columbia University, New York, New York 10027, United States

[6]Department of Materials Science and Engineering, Korea Advanced Institute of Science and Technology (KAIST), Daejeon, 34131, Republic of Korea

[7]Department of Electrical and Electronic Engineering, University of Melbourne, Parkville, Victoria 3010, Australia

[8]School of Chemistry, University of Melbourne, Parkville, Victoria 3010, Australia

[9]Department of Quantum Science and Technology, Research School of Physics, The Australian National University, Canberra, Australia

[10]The Molecular Foundry, Division of Molecular Biophysics & Integrated Bioimaging, Lawrence Berkeley National Laboratory, Berkeley, California 94720, United States

*kenneth.crozier@unimelb.edu.au

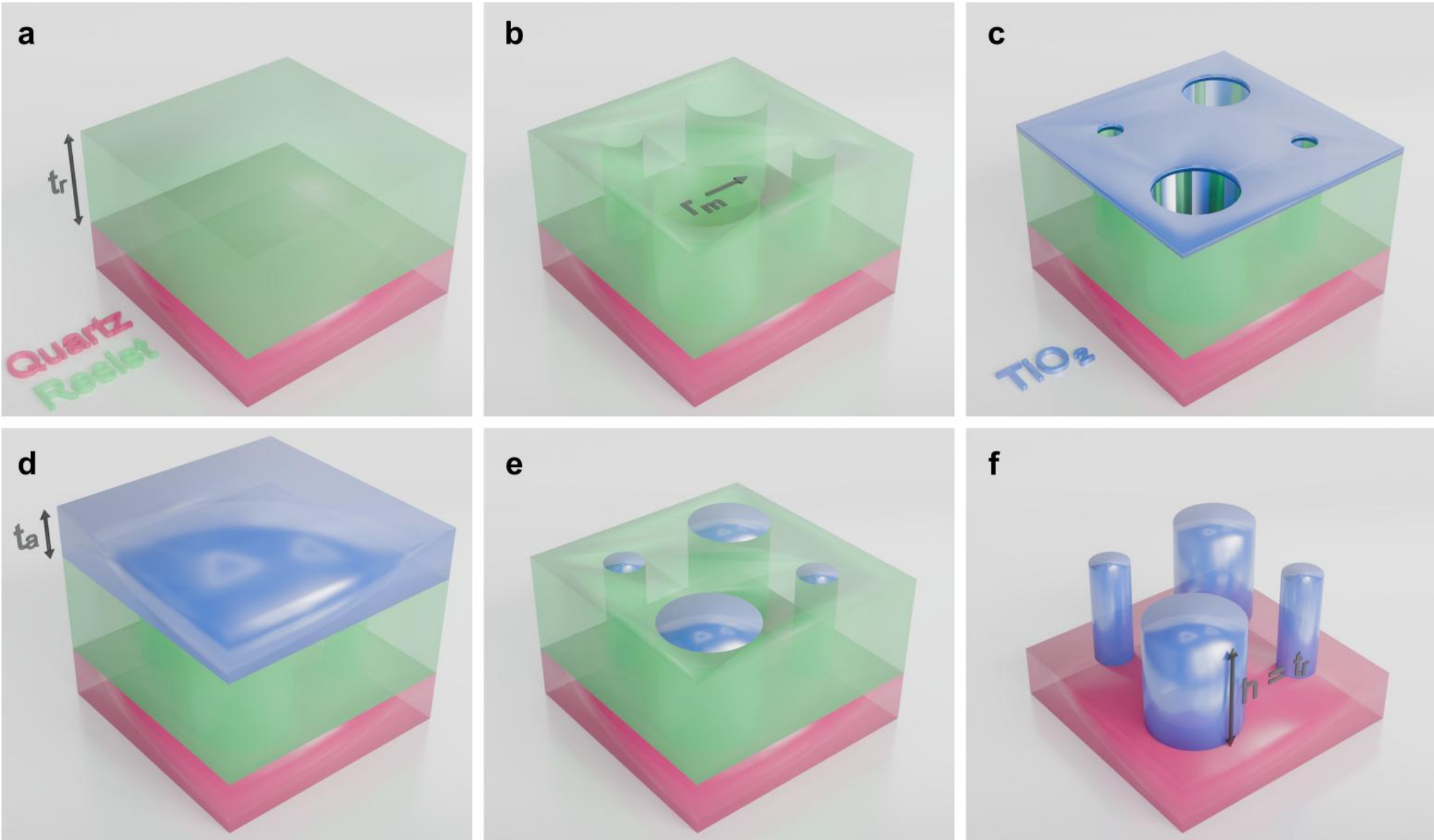

Fig. S1 Fabrication process of the dielectric metasurface. **a** Electron-beam resist with thickness t_r is spin-coated onto a quartz substrate, followed by a thin conductive polymer layer (not shown). **b** Resist patterned by electron-beam lithography (EBL), where r_m is the maximum opening radius. **c** Initial TiO_2 deposition via atomic layer deposition (ALD), conformally coating the resist. **d** TiO_2 deposition completed to thickness t_a, ensuring $t_a \geq r_m$. **e** Reactive ion etching (RIE) of TiO_2 to expose the resist. **f** Final dielectric metasurface with height t_r after resist removal.

Table S1: Particle swarm optimisation parameter ranges and constraints

Parameter	Range/constraint
p	400 to 1200 nm
r_i, i = 1 to 4	0 to 200 nm, $r_3 = r_4$
h	100 to 375 nm
t	h + 50 nm
Centre of unit cell	(x, y) = (0,0)
Centre of pillars	(x, y) = ± (p/4, p/4)
Generation size	400

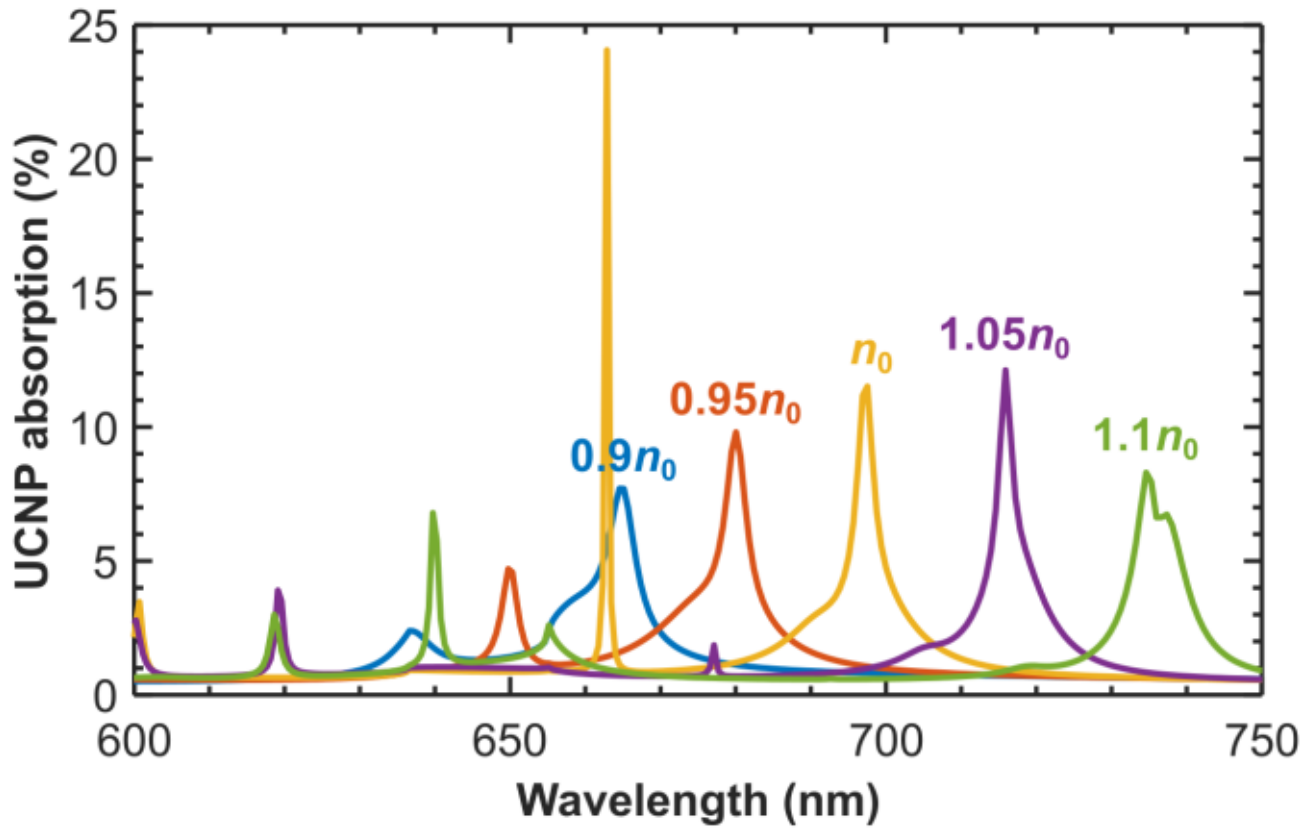

Fig. S2 Sensitivity of the emission band resonance to the refractive index of the UCNP film. Simulated UCNP light absorption for varying refractive index values. n_0 denotes the refractive index value of the UCNP film used for metasurface design.

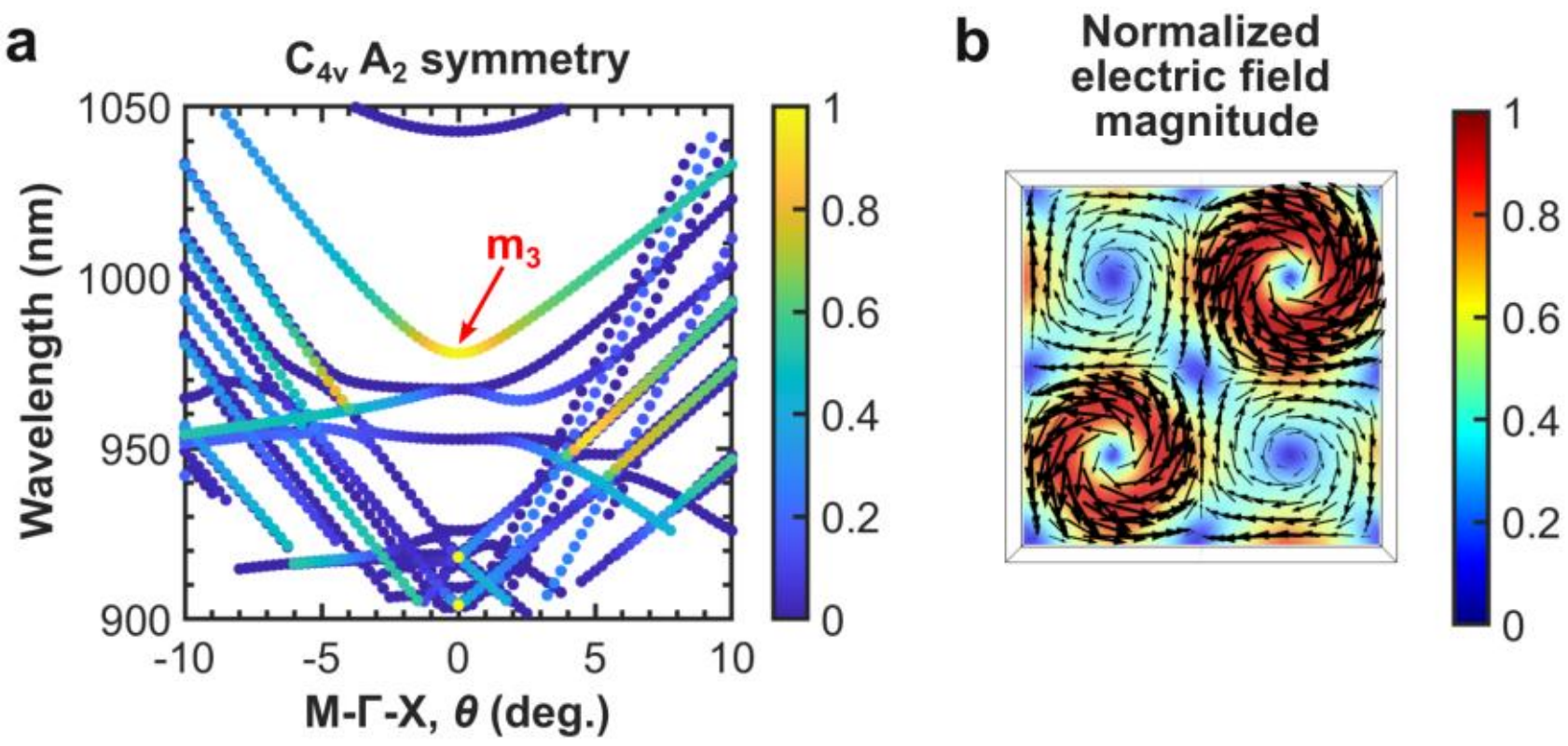

Fig. S3 Optical mode analysis of the UCNP-coated dielectric metasurface. a Symmetry analysis of the metasurface modes around the UCNP absorption band with respect to the C_{4v} A_2 irreducible representation, highlighting dark modes. **b** Electric field distribution of the dark mode labelled m_3 in panel a on a horizontal plane intersecting the mid-height of the nanopillars.

Table S2: Upconversion enhancement provided by various structures

Optical structure	Excitation/Emission wavelength (nm)	Excitation Intensity (W cm^{-2})	Enhancement	Ref.
Dielectric	976/660	15.9	1100	This work
Dielectric	1550/660	35	2400	39
Plasmonic	972/654	120	1350	31
Plasmonic	980/540	20	1198	28
Dielectric	980/660	7.5	500	38
Dielectric	965/800	Not reported	400	42
Dielectric	980/660	10	350	40
Dielectric	980/540	92	51	36
Plasmonic	980/660	1000	39	33
Dielectric	1550/980	2.8	2.7	37

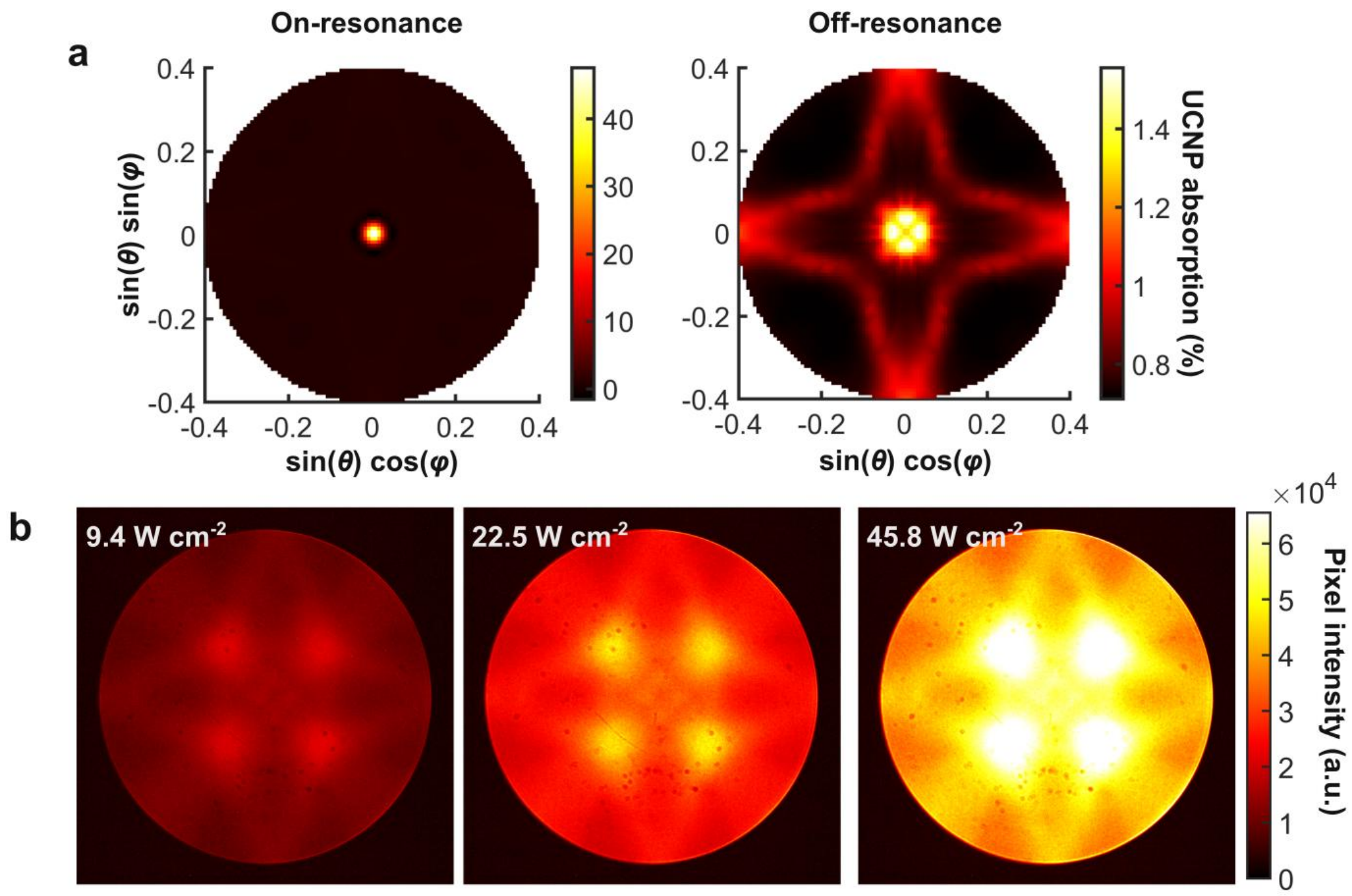

Fig. S4 Angular distribution of light emission from the metasurface-UCNP screen. a Simulated light absorption in the UCNP film as a function of incident angle on the emission band resonance (λ = 660 nm) and nearby this resonance. **b** Experimental back focal plane images captured using a 0.4 NA objective lens, illustrating the angular distribution of red light emission under varying excitation intensities at λ = 976 nm.

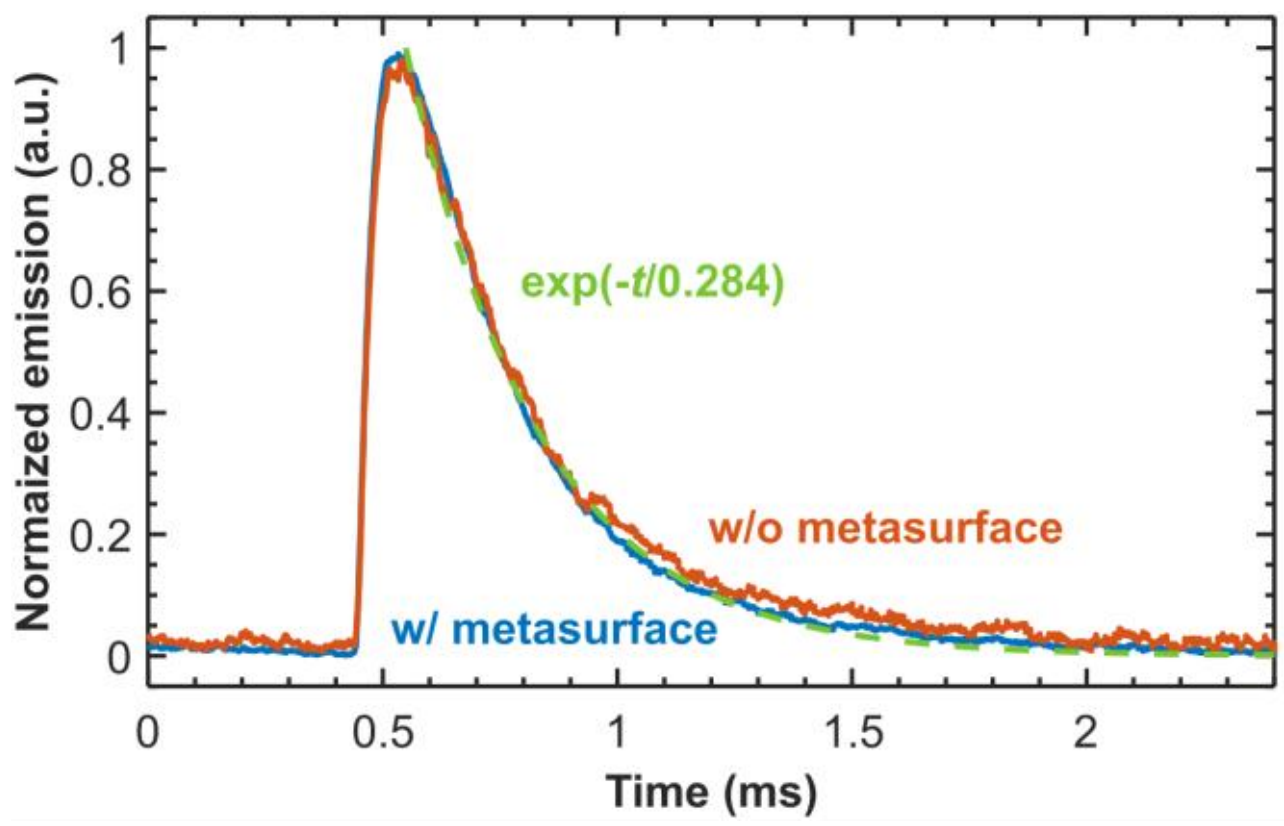

Fig. S5 Emission decay kinetics under pulsed excitation. Time-resolved emission of UCNPs with and without the metasurface under λ = 980 nm excitation with a pulse width of 1.3 μs.

Edge-based modulation transfer function (MTF) analysis

To quantitatively evaluate spatial resolution preservation in the metasurface–UCNP screen, an edge-based modulation transfer function (MTF) analysis was performed on both the visible reference image and the upconverted image recorded at the camera plane. The analysis was performed on two independent edge regions to ensure reproducibility, yielding consistent results; one representative example is shown in Fig. S6.

Measured MTF Performance: The image was formed using a 20× / 0.4 NA objective lens. Spatial frequencies therefore scale with magnification according to $f_{screen} = M \times f_{camera}$, where $M = 20$. The extracted MTF50 values at the camera are ~0.01 cycles μm^{-1} for the visible image and ~0.005 cycles μm^{-1} for the upconverted image. Converting to the metasurface (screen) plane, MTF values are 0.01 × 20 = 0.20 cycles μm^{-1} for the visible and 0.005 × 20 = 0.10 cycles μm^{-1} for the upconverted images. This indicates a reduction of high-spatial-frequency contrast by approximately a factor of two after upconversion. Importantly, MTF50 does not represent a hard resolution cutoff. Rather, it denotes the spatial frequency at which image contrast drops to 50% of its low-frequency value. Higher spatial frequencies may still be transmitted with reduced contrast. Spatial frequencies associated with ~20 μm features of the object (0.05 cycles μm^{-1}) lie below the measured upconverted bandwidth (~0.10 cycles μm^{-1}), explaining the preservation of these features in the infrared-to-visible images.

Detector Sampling Limit: The camera used in this study is a colour CMOS sensor with a pixel pitch of 4.54 μm. After 2×2 Bayer averaging, the effective pixel pitch becomes: 2 × 4.54 = 9.08 μm, corresponding to a Nyquist spatial frequency at the camera plane of 1 / (2 × 9.08) = 0.055 cycles μm^{-1}. Accounting for magnification, the detector-limited Nyquist frequency at the metasurface plane is 20 × 0.055 = 1.1 cycles μm^{-1}. Since this value is substantially higher than the measured MTF50 values, detector sampling does not limit the spatial resolution at the metasurface plane.

Theoretical Cutoff from ±6° Flat Band: Angle-resolved measurements show that the metasurface resonance remains nearly invariant within approximately ±6°. The corresponding effective numerical aperture is $NA_{eff} = \sin(6°) = 0.105$. The maximum spatial frequency supported by this angular bandwidth at the emission wavelength (λ = 0.66 μm) is $f_{cutoff} = NA_{eff} / \lambda = 0.105 / 0.66 = 0.159$ cycles μm^{-1}. This value is defined at the metasurface plane. The measured upconverted MTF50 (0.10 cycles μm^{-1}) lies below the theoretical ±6° cutoff, indicating consistency between the experimentally observed bandwidth and the metasurface angular response.

Role of the 20× / 0.4 NA Objective: The diffraction-limited cutoff frequency of the objective lens is $f_{cutoff} = NA / \lambda = 0.4 / 0.66 = 0.61$ cycles μm^{-1}. This value is significantly larger than both the metasurface angular cutoff (~0.159 cycles μm^{-1}) and the measured upconverted MTF50 (0.10 cycles μm^{-1}). Therefore, the objective lens does not limit the spatial resolution in the present system.

Contribution of the Relay Optical System: The imaging path includes relay optics between the object and the metasurface. The total system MTF can be expressed as $MTF_{total} = MTF_{relay} \times MTF_{metasurface} \times MTF_{objective} \times MTF_{detector}$. Since the relay optics are common to both visible and upconversion measurements but operate at different wavelengths (visible vs. 980 nm excitation), wavelength-dependent aberrations and alignment tolerances may contribute to additional degradation in the upconverted case. Consequently, part of the observed reduction in MTF50 may originate from relay optics rather than solely from the metasurface angular response.

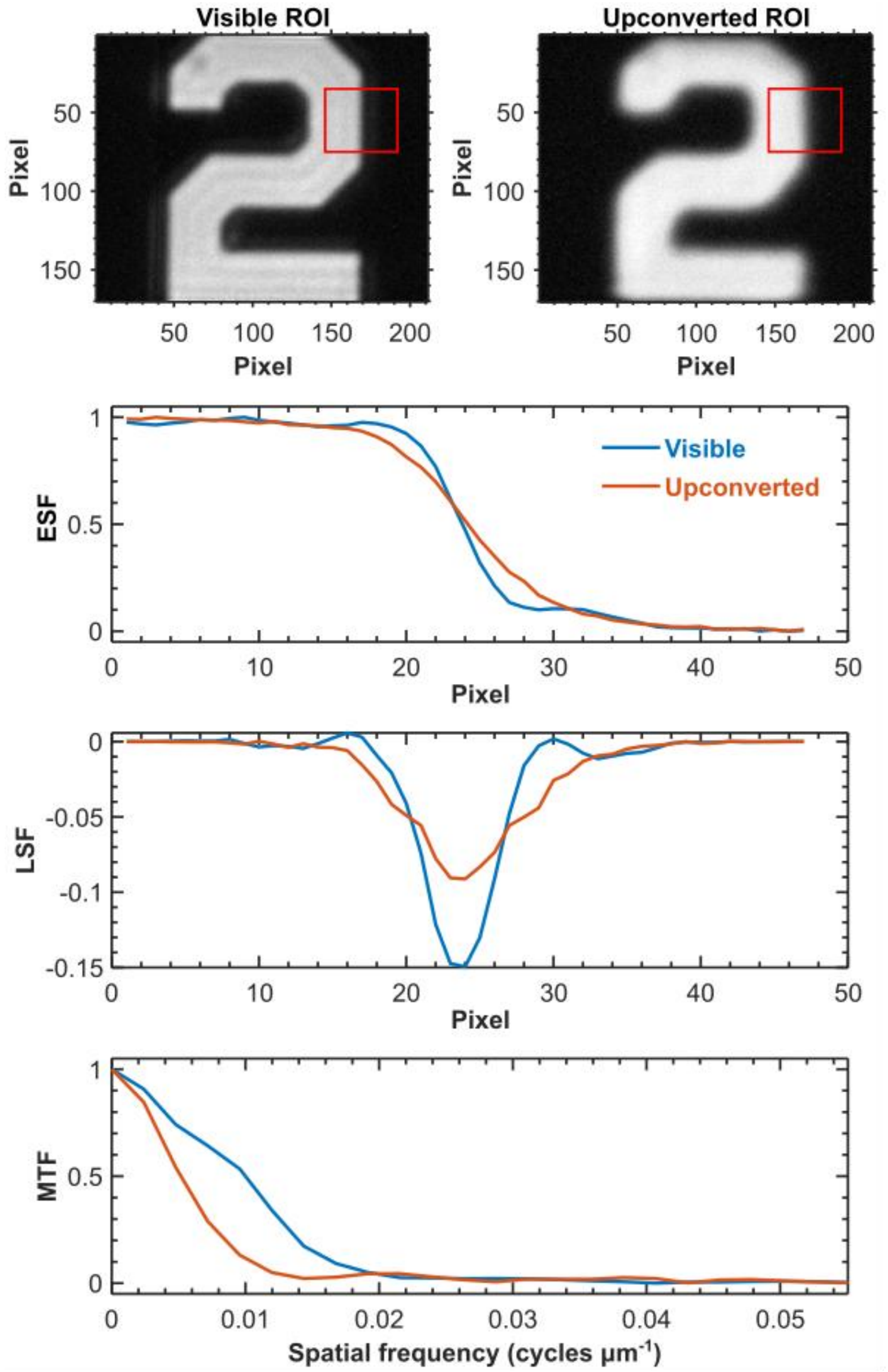

Fig. S6 Edge-based modulation transfer function (MTF) analysis of visible and upconverted images. The visible reference image and the infrared-to-visible upconverted image are shown with the selected region of interest (ROI) indicated by a red box for MTF evaluation. From the extracted ROI, the normalized edge spread function (ESF) is obtained and differentiated to yield the line spread function (LSF). The Fourier transform of the LSF provides the corresponding MTF curves for both visible and upconverted cases, enabling quantitative comparison of spatial frequency preservation after upconversion.

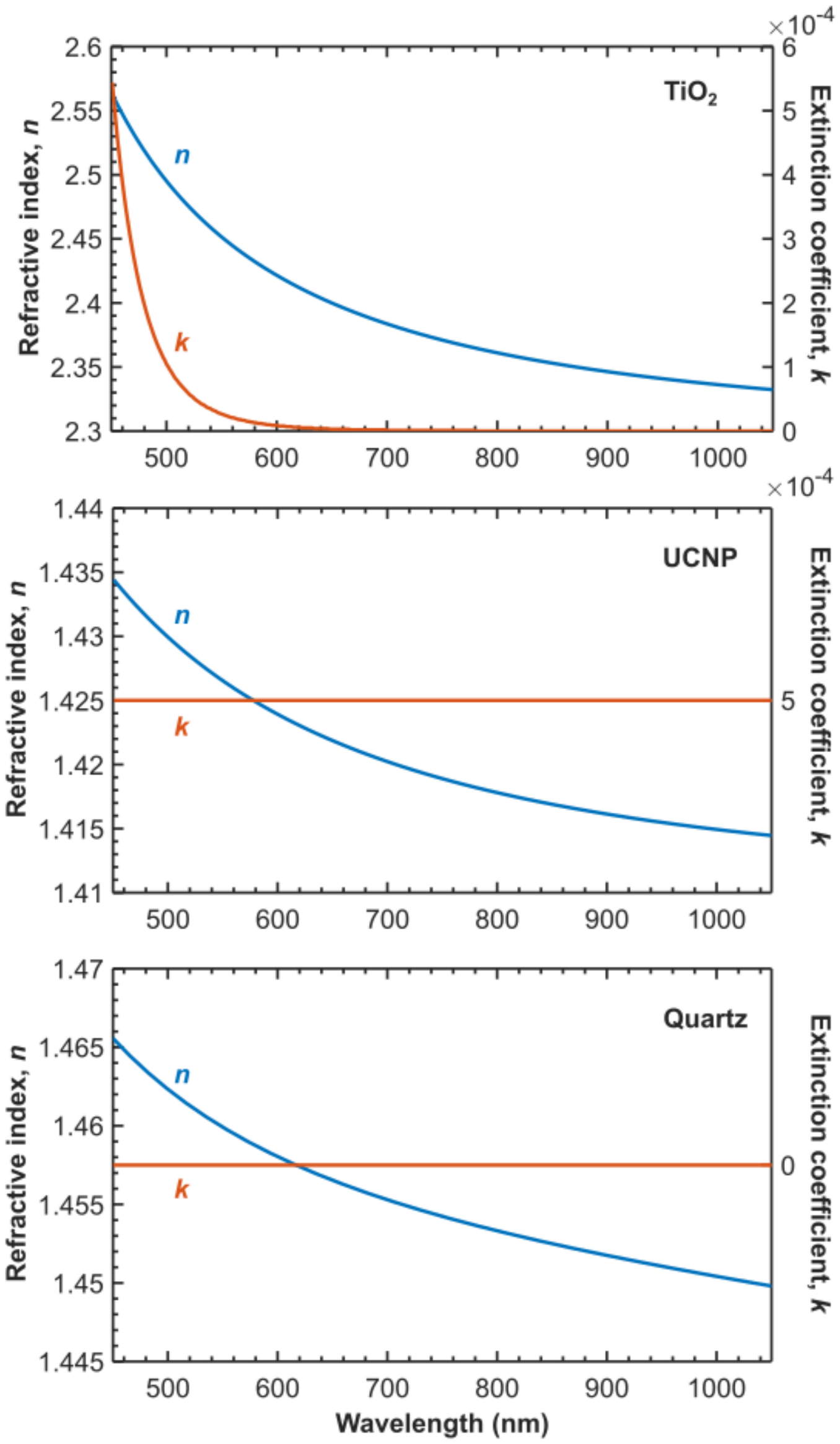

Fig. S7: Refractive index values of different materials used in optical simulations as a function of wavelength.

Synthesis of upconverting nanoparticles

Materials. Ytterbium (III) chloride ($YbCl_3$, 99.9%), erbium (III) chloride ($ErCl_3$, 99.9%), yttrium (III) chloride (YCl_3, 99.99%), ammonium fluoride (NH_4F, 99.99%), oleic acid (OA, 90%), and 1-octadecene (ODE, technical grade, 90%), and sodium trifluoroacetate (CF_3COONa, 98%) were purchased from Sigma Aldrich. Sodium oleate (>97.0%) was purchased from TCI Chemicals. All chemicals were used without further purification.

Synthesis of alloyed $NaYb_{0.8}Er_{0.2}F_4$ core nanocrystals. $YbCl_3$ (0.8 mmol, 223.5 mg), $ErCl_3$ (0.2 mmol, 54.7 mg), oleic acid (12 mL, 10.74 g), and 1-octadecene (ODE, 12 mL) were added to a 100 mL round bottom flask with a stir bar. The flask was placed under vacuum and heated to 110 °C for 1 h to remove H_2O and O_2. Once the solution became clear, the flask was filled with N_2 and cooled to room temperature (RT). Sodium oleate (4 mmol, 1217 mg) and NH_4F (4 mmol, 148.2 mg) were added, and the flask was again placed under vacuum, stirred at RT for 20 min, and flushed three times with N_2. The reaction was then heated to 320 °C for 30 min under N_2, followed by cooling to 60 °C with a stream of air after removal of the heating mantle.

To isolate the nanoparticles, the suspension was transferred to a 50 mL centrifuge tube, and EtOH (20 mL) was added before centrifugation at 4000 rpm for 5 min. The supernatant was discarded, and the pellet was suspended in 5 mL of hexane by vortexing. The solution was centrifuged again at 4000 rpm for 2 min to remove larger particles and aggregates. The supernatant was transferred into another 50 mL centrifuge tube and washed two additional times by adding EtOH. The supernatant was discarded, and the nanocrystals were resuspended and stored in hexane.

Precursor solutions for shell growth. A 0.10 M yttrium oleate (Y-OA) solution was prepared by adding anhydrous YCl_3 (1 mmol, 195 mg) into a mixture of 4 mL oleic acid and 6 mL ODE in a 50 mL three-neck flask and heating to 110 °C under vacuum for 1 h, producing a clear solution. In a separate flask, sodium trifluoroacetate (2 mmol, 272 mg) was added to a mixture of 4 mL oleic acid and 6 mL ODE, then heated to 50 °C under vacuum until a clear solution formed, giving a 0.20 M Na-TFA-OA shelling solution. Both precursor solutions were cooled and transferred into a glovebox for shell growth.

Shell growth. To grow ~4 nm inert $NaYF_4$ shells on 14 nm $NaYb_{0.8}Er_{0.2}F_4$ nanocrystals (resulting in a final diameter of 22 nm), 40 mg of OA-capped nanocrystals in hexane were pipetted into a 40 mL vial, and the solvent was evaporated using a gentle N_2 flow at room temperature. The vial was transferred into the WANDA glovebox, and degassed ODE (6 mL) and OA (4 mL) were added, along with two stir bars. The reaction vial and precursor vials were loaded onto the robot deck, and the automated shell growth procedure was initiated. During the procedure, the reaction vial was heated to 280 °C. Once the temperature stabilized, 8 SILAR cycles were performed, each consisting of a Y-OA injection followed by a Na-TFA-OA injection, with volumes listed in Table S3. Injections were spaced 20 min apart, and the reaction temperature was maintained at 280 °C throughout and for 20 min after the final injection. The reaction was then rapidly cooled under N_2 flow. The nanocrystals were washed using the same procedure described for the cores.

Table S3: Injection volumes of precursor solutions for controlled growth of $NaYF_4$ inert shells on alloyed $NaYb_{0.8}Er_{0.2}F_4$ nanocrystals.

Injection cycle	1	2	3	4	5	6	7	8
Y-OA (mL)	0.361	0.412	0.465	0.523	0.583	0.647	0.714	0.784
Na-TFA-OA (mL)	0.361	0.412	0.465	0.523	0.583	0.647	0.714	0.784

UCNP spin-coating

Uniform metasurface–UCNP screens were fabricated as follows. Dried UCNP powder was redispersed in toluene after hexane was evaporated under a nitrogen flow. The dispersion was vortexed for several minutes, sonicated for 1 h to ensure uniform suspension, and then filtered through a 0.45 µm PTFE filter. The concentration of the filtered UCNP suspension—which determined the thickness of the spin-coated film—was tuned either by further nitrogen blowing (to concentrate) or by adding toluene (to dilute). Immediately before spin coating, the metasurface chips were surface treated by rinsing with toluene, and the UCNP suspension was briefly sonicated for 2 min to redisperse any aggregates. For spin coating, a 30 µL droplet of the suspension was deposited onto the (12.5 × 12.5 mm) chip and spread using a two-step process: (i) 700 rpm, 100 rpm s^{-1}, 20 s; (ii) 3000 rpm, 500 rpm s^{-1}, 60 s.

To calibrate film thickness versus solution concentration, UCNP suspensions were prepared at 30 mg mL^{-1} and progressively concentrated by solvent evaporation. At each step, a bare quartz substrate was spin-coated, the resulting film was scratched with a sharp tip, and the step height was measured by atomic force microscopy (AFM). A concentration of ~240 mg mL^{-1}—approximately one-eighth of the initial volume—yielded the target film thickness of ~425 nm. The root-mean-square surface roughness (Rq) measured over multiple areas ranged from 4–6 nm, indicating good thickness uniformity and optical smoothness. The roughness is significantly smaller than the optical wavelengths of interest (660–975 nm), suggesting negligible scattering contribution from surface irregularities.

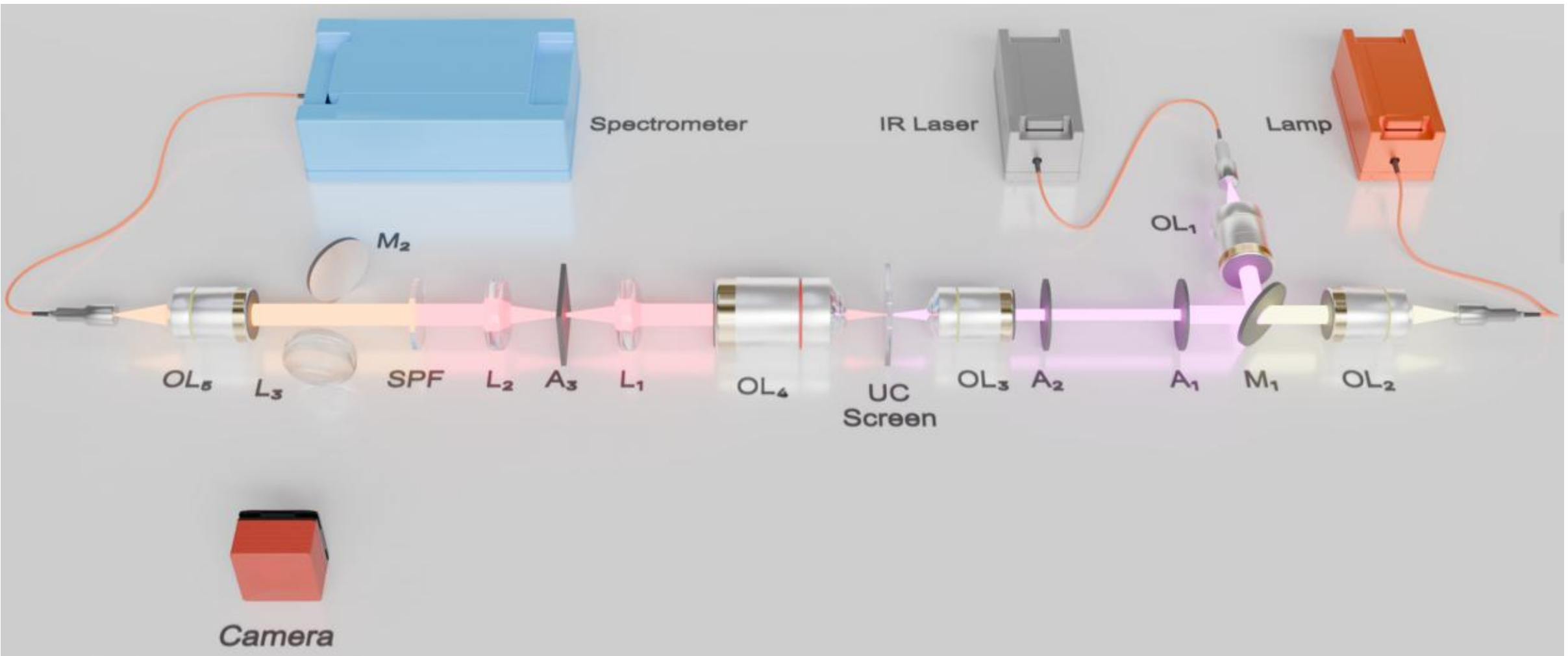

Fig. S8 Schematic of the experimental setup used for optical transmission and upconversion measurements.

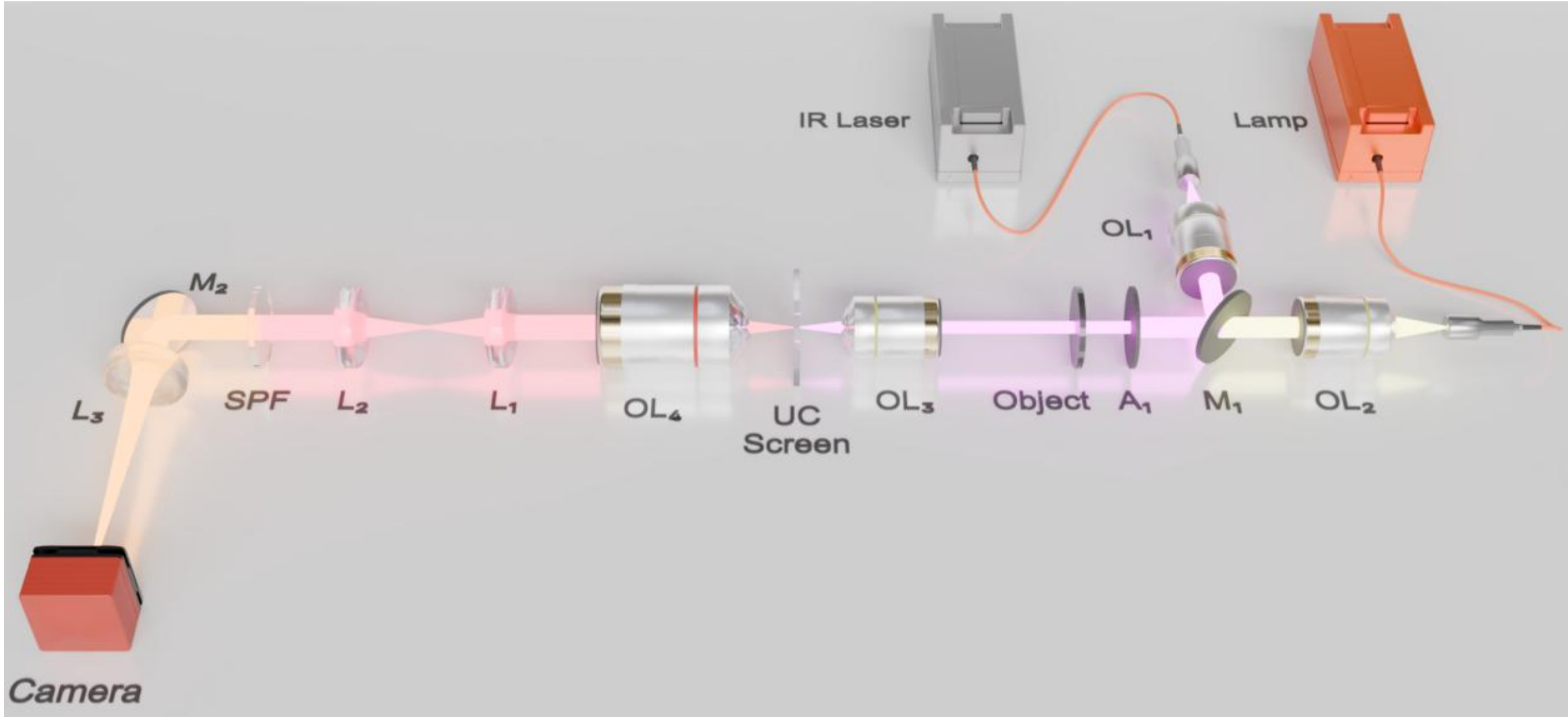

Fig. S9 Schematic of the experimental setup used for upconversion imaging.

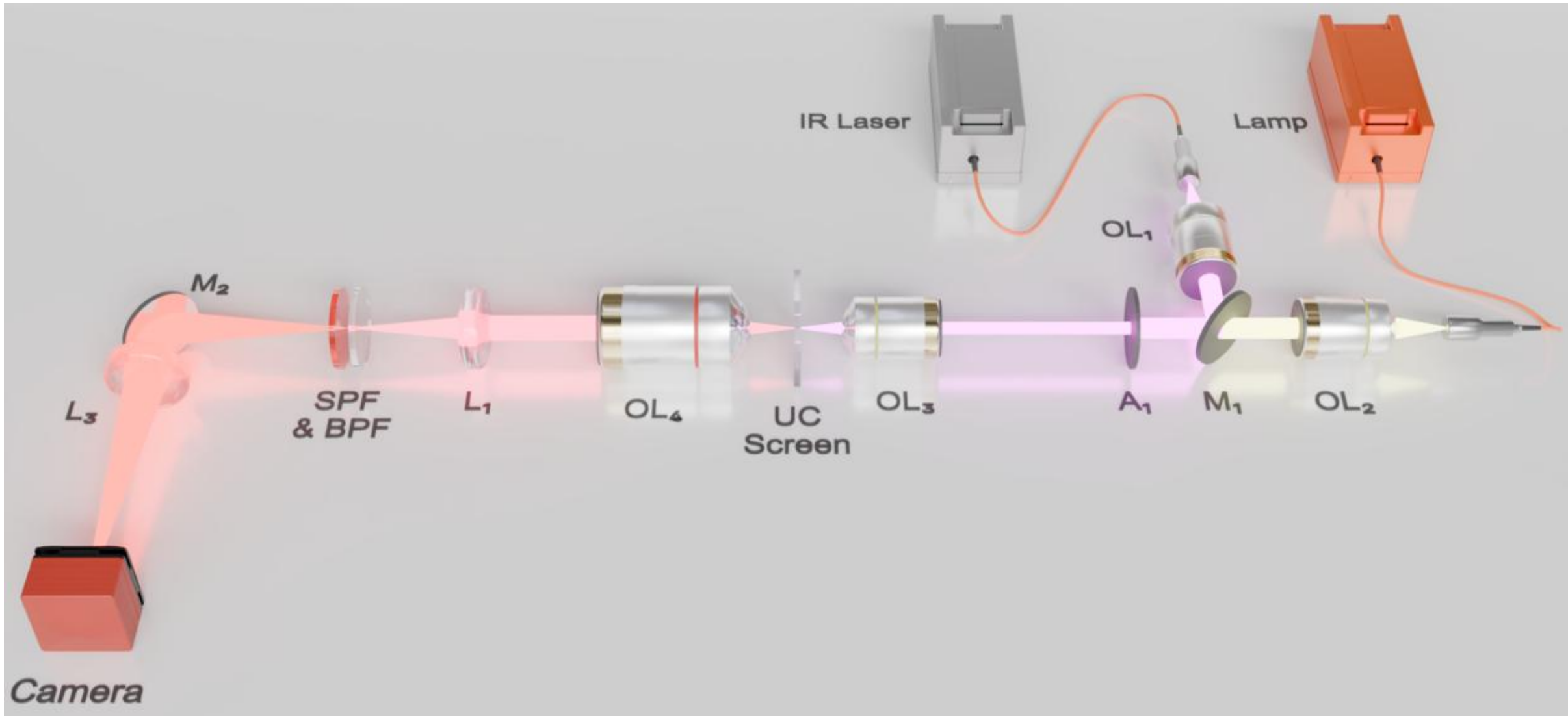

Fig. S10 Schematic of the experimental setup used for back focal plane imaging.
